# Efficacy of prescription-eligible digital health applications for depression and generalized anxiety disorder in Germany: a systematic review and meta-analysis

**DOI:** 10.1038/s41598-026-68875-y

**Published:** 2026-09-11

**Authors:** A. Schmitz, S. Frey, A. Karimzadeh, A. L. Neves, A. Mayr, B. Weltermann

**Affiliations:** 1 Institute for Family Medicine, University of Bonn, University Hospital Bonn, Bonn, Germany; 2 Department of Primary Care and Public Health, Imperial College London, London, UK; 3 Department of Medical Biometry and Statistics, University of Marburg, Marburg, Germany

**Keywords:** Diseases, Health care, Medical research, Psychology, Psychology

## Abstract

In Germany, prescription-eligible digital mental health applications (DiGA) were introduced in 2020 as promising interventions to address, among others, depression and anxiety disorders, two of the most prevalent mental health conditions worldwide. Despite growing interest in DiGAs, their overall efficacy remains uncertain. This study aimed to systematically evaluate and quantify the efficacy of prescription-eligible digital interventions for depression and generalized anxiety disorder by synthesizing evidence from randomized controlled trials (19 trials; total *N* = 4,078; pooled mean age = 38.7 years, SD = 12.1). Here we show that prescription-eligible digital applications for depression and generalized anxiety disorder reduce symptom severity compared with control conditions. For depression, effects were observed both immediately after the intervention (number of apps = 5; k = 17; SMD = − 0.49; 95% CI − 0.65 to − 0.32) and at follow-up (number of apps = 1; k = 4; SMD = − 0.35; 95% CI − 0.46 to − 0.29), while evidence for generalized anxiety disorder was limited due to a small number of available studies (number of studies = 2). These findings support the integration of evidence-based digital tools into mental health treatment strategies in Germany. However, the available evidence is currently dominated by a small number of applications, particularly Deprexis, and should therefore not be interpreted as equally representative of all DiGAs currently listed for depression in Germany. The findings also highlight methodological limitations of current research and underscore the need for real-world evaluations, which address not only efficacy but also the effectiveness, content, quality and implementation.

**Supplementary Information:**

The online version contains supplementary material available at https://doi.org/10.1038/s41598-026-68875-y.

## Introduction

Anxiety Disorders and Depression are among the leading global causes of disease burden, affecting individuals across all age groups and imposing substantial personal, societal and economic costs. Worldwide, an estimated 280 million people suffer from depression, corresponding to a global prevalence of 3.6%. Prevalence increases with age and is highest among individuals over 60 years of age^1,2^. In Germany, depression represents a major public health concern, with 16.7% of adults diagnosed in outpatient care in 2023^3^. Women are more frequently affected than men (20.2% vs. 12.6%)^3^.

The economic impact of depression is considerable. A recent review by Eden et al. (2021) estimates annual per-patient costs up to €3,000 depending on study population and cost definitions^4^. Based on analyses of the German Health Interview and Examination Survey for Adults (DEGS), adults with medically diagnosed depression incurred adjusted annual costs of approximately €9,539 compared with €4,492 among matched adults without depression, corresponding to excess costs of approximately €5,047 per affected individual. More than half of these excess costs were attributable to indirect costs resulting from sick leave and early retirement (€2,835), exceeding direct healthcare costs €2,212^5^. These calculations do not reflect the substantial intangible burden, including reduced quality of life and life expectancy.

Alongside depression, anxiety disorders, including generalized anxiety disorder (GAD), are the most prevalent mental health disorders globally, affecting approximately 301 million people in 2019^6,7^. Among anxiety disorders, GAD is one of the most common and persistent conditions and is frequently comorbid with depression^8^. In Germany, 7.9% of adults were diagnosed with an anxiety disorder in 2023; similar to depression, women were more frequently affected than men (9.9% vs. 5.4%). Higher rates of anxiety disorders are prevalent in younger persons (ages 15–19: 11.16%; ages 20–24: 9.98% vs. age-standardized: 7.6%)^9^.

Despite the high burden of disease, access to guideline-based treatment remains limited. Germany continues to face a limited timely access to evidence-based mental healthcare and long waiting times for psychotherapy. According to a an analysis of statutory health insurance claims data, 61% of adults waited longer than the recommended 14 days for an acute treatment appointment in 2024, with an average waiting time of approximately 42 days^10^. This shortage affects individuals with mild to moderate depression as well as patients with anxiety disorders, who are at risk of delayed care despite their excellent prognosis when treated in a timely manner.

Given the limited capacity of traditional care systems, digital health applications (DiGA – *Digitale* *Gesunheitsanwendungen*) have emerged as a low-threshold, scalable intervention for treating various diseases. In Germany, DiGAs can either be prescribed by physicians or psychotherapists or obtained directly from the health insurance providers upon proof of an eligible diagnosis. In both cases, DiGAs are reimbursed by the statutory health insurance. Following prescription or approval of a direct application, patients receive an activation code through their health insurance provider, enabling immediate access to the application.

Germany is a pioneering country in the field of DiGAs, where they are regulated by the Digital Healthcare Act (DVG – *Digitales-Versorgungs-Gesetz*). The law establishes the so-called “fast-track” process, which provides a unique opportunity for the rapid implementation of DiGAs into routine care. Within this process, DiGAs can be provisionally provided if preliminary evidence suggests a potential positive care effect (e.g. through literature), allowing reimbursement during the evaluation period. Following the evaluation period, applications remain permanently listed only if sufficient evidence for positive healthcare effects has been demonstrated. Otherwise, they are removed from the DiGA directory managed by the Federal Institute for Drugs and Medical Devices^11^. However, the supporting evidence must be carefully contextualized and interpreted within this accelerated framework. The immediate availability and flexible integration into everyday life are major benefits of digital interventions. This is particularly relevant for patients with mental illness, when stigma and feelings of shame may hinder seeking conventional care^12^. Digital mental health interventions have been extensively evaluated and are supported by a growing number of evidence. Recent meta-analyses have demonstrated small to moderate beneficial effects for digital interventions in the treatment of depression, although effect estimates vary depending on intervention characteristics and comparator conditions^13,14^. Against this background, DiGAs represent a promising approach to delivering evidence-based mental health care.

Despite the growing number of DiGAs listed for depression and GAD, the evidence regarding their efficacy remains limited. Previous studies and systematic reviews have reported beneficial effects for digital therapeutics and DiGAs, but findings vary across interventions and populations and concerns regarding risk of bias remain^15–19^. Moreover, no existing review has comprehensively evaluated all currently listed DiGAs for depression and GAD in terms of clinical efficacy. A DiGA-specific synthesis is warranted because these interventions are evaluated and reimbursed within a standardized German regulatory framework that differs from the broader category of digital therapeutics.

This systematic review and meta-analysis therefore evaluated the efficacy of permanently listed DiGAs in reducing symptoms of depression and GAD compared with control conditions and assessed the risk of bias of the included randomized controlled trials (RCTs).

## Results

### DiGA identification and selection

**Fig. 1:**
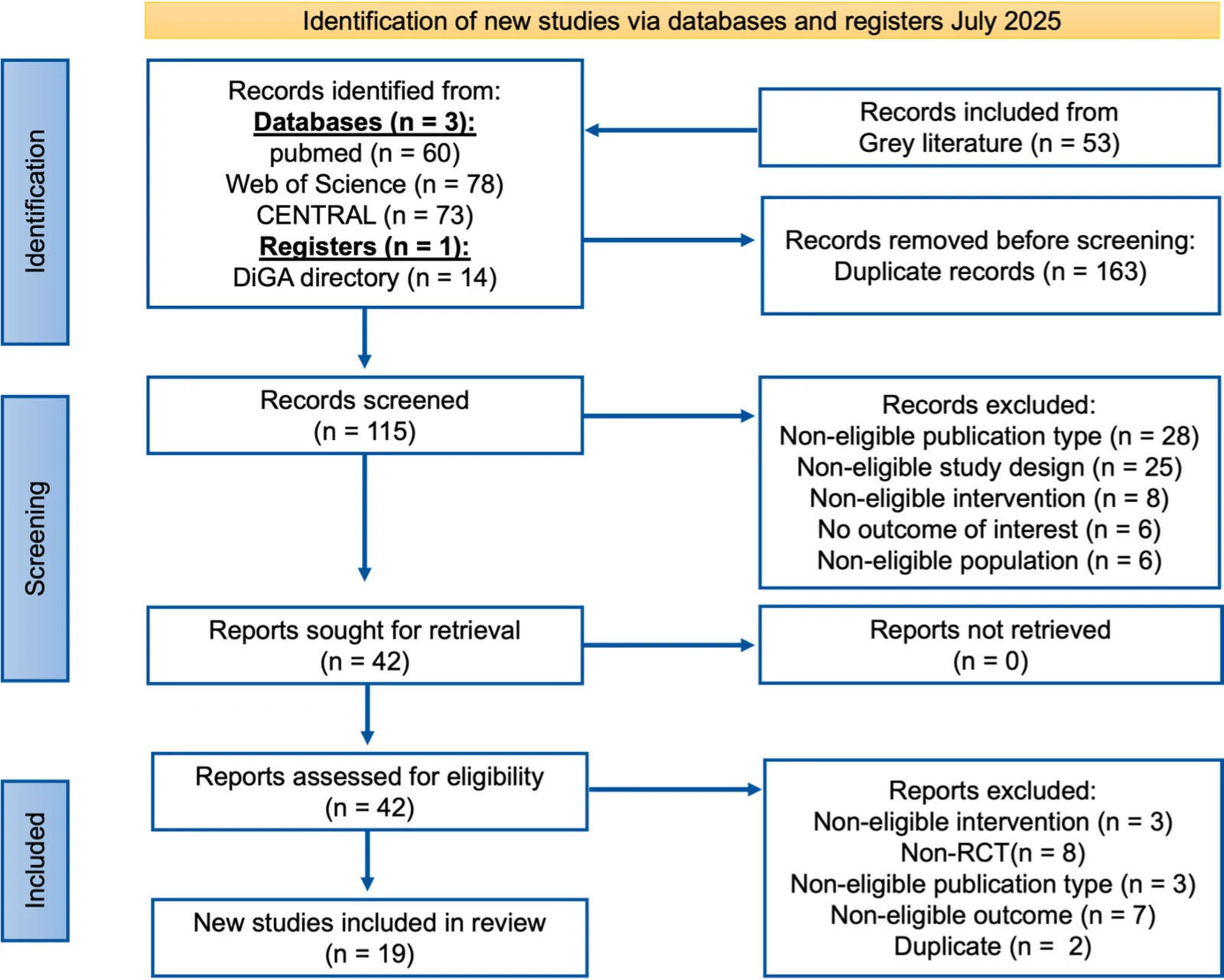
PRISMA flow chart for Study Identification & Selection. PRISMA flow chart of the process of identification, screening and selection of studies. n, number of studies.

A total of 57 DiGAs were identified through a systematic search of the official DiGA directory^20^. Of these, 38 were excluded due to either not having a permanent listing status or targeting an unrelated health domain. Following eligibility assessment, eight DiGAs targeting depression or GAD met the inclusion criteria and were included in the review^21^. These were *Deprexis* (GAIA AG), *edupression.com* (SOFY GmbH), *elona therapy Depression* (Elona Health GmbH), *My7steps App* (My7steps GmbH), *Novego: Depression bewältigen* [Novego: Coping with depression] (IVPNetworks GmbH), *Selfapys Online-Kurs bei Depression* [Selfapys online course for depression] (Selfapy GmbH), *Selfapys Online-Kurs bei Generalisierter Angststörung* [Selfapys online course for generalized anxiety disorder] (Selfapy GmbH) and *velibra (*GAIA AG). DiGAs are generally prescribed for an initial period of 90 days and may be extended if clinically indicated. The included DiGAs use some form of CBT (detailed information about the contents can be found in the DiGA directory)^20^. The identification and selection process is illustrated in Supplementary Material S1 Fig. 1.

### Study identification and selection

Studies were selected according to the predefined eligibility criteria described in the Methods section. A total of 278 records were initially identified. Of these, 211 records were retrieved through systematic database searches in Pubmed, Web of Science and Cochrane CENTRAL, while an additional 67 records were identified through register and grey literature searches (including manufacturer websites). After removing 163 duplicates, 115 records remained for title and abstract screening. Of these, 73 were excluded, as they did not meet the predefined inclusion criteria. Subsequently, 42 full-text articles were assessed for eligibility. Following full-text review, 19 studies met all eligibility criteria. An additional four studies were considered for eligibility: Three of these were excluded due to the absence of an appropriate intention-to-treat (ITT) population using any kind of imputation^22–24^. One study was a follow-up publication of a trial that had already been included in the analysis; it was therefore incorporated into the follow-up evaluation accordingly^25^.

The study selection process was independently conducted by two reviewers (SF and AS). Inter-rater agreement was substantial during both title/abstract screening (Cohen’s κ = 0.79, *p* < 0.001) and full-text screening (Cohen’s κ = 0.73, *p* < 0.001). Any discrepancies were resolved through discussion, led by a third reviewer (AK). The complete identification and selection process is illustrated in Fig. 1.

### Summary of the characteristics of the included studies

*Participant Characteristics.* A total of 19 RCTs including 4,078 participants were analyzed. The study characteristics are summarized in Table 1. The majority of studies (42.1%) had sample sizes between 101 and 200 participants, 15.79% of the studies included over 300 participants, 26.3% enrolled 51 to 100 participants. Participants in the intervention group had a weighted mean age of 38.7 years (SD = 12.1). In 14 studies, no comorbid diagnosis were reported. Five studies examined participants with comorbid conditions, including epilepsy, multiple sclerosis, schizophrenia, or self-reported problems with gambling or pain symptoms. Although reporting on participants’ educational background was heterogeneous across studies, it was evident that the majority had comparatively high levels of education (often with an academic degree).

*Contextual Characteristics.* Most of the studies were conducted within the German health care context. Germany alone accounted for 15 out of 19 studies (78.9%). Two studies (10.5%) were conducted across Switzerland, Germany, and Austria. One study was conducted in the United States and one in Brazil (5.2% each). The majority of studies were conducted in an outpatient setting (89.4%). At baseline, 28.94% of patients in the intervention groups and 31.82% in the control groups were taking medication, and 18.06% and 19.91%, respectively, were receiving psychotherapy.

*Intervention Characteristics.* The vast majority of studies (*n* = 17, 89.5%) assessed interventions for depression, while only two studies focused on DiGAs for GAD. The most frequently evaluated DiGA was *Deprexis* (12 out of 19 studies; 63.1%). Other studies included the DiGAs *Novego: Depressionen bewältigen* (*n* = 3; 15.7%), *Selfapys Online-Kurs bei Depression* (*n* = 1; 5.2%), *Selfapys Online-Kurs bei Generalized Anxiety Disorder* (GAD) (*n* = 1; 5.2%), *velibra* (*n* = 1; 5.2%) and *elona therapy* *Depressionen* (*n* = 1; 5.26%). No eligible published peer-reviewed RCT was identified for *edupression.com* or the *My7steps App*. While permanently listed DiGAs are required to provide evidence of a positive healthcare effect to the BfArM, supporting studies are not necessarily available as peer-reviewed publications.

*Study Design Characteristics.* All studies included in the analysis were RCTs. The studies were published between 2009 and 2024. Nine studies (47.3%) were published from 2009 to 2016, four studies (21.0%) in 2017, and six studies (31.5%) from 2018 onward. All studies reported post-intervention outcomes and almost half reported follow-up data (*n* = 9; 47.3%). Manufacturer involvement varied across the included studies. At a minimum, the respective DiGA was provided by the manufacturer for study participants. In several studies, additional forms of involvement were reported, including manufacturer affiliation of authors, study funding or sponsorship, and financial conflicts of interest. Further study characteristics including the involvement of manufacturers, duration of intervention, outcome measures, follow-up periods, and randomization methods are detailed in Supplementary Material S2.

**Table 1:** Studies included in the Review and Meta-analysis.

Study (year)	DiGA (duration)	Study Population Symptoms; Age Range; Comorbidities; Group with mean age (SD) and % female	Study Design: IG, *n*; type of CG, *n*	Primary Outcome (Measurement)	Drop-outs	Results at post-intervention
Miegel et al. (2019)^26^	Novego (12 weeks)	*depressive symptoms*: no DSM-V diagnosis required, subjective assessment by the participant *age*: between 18 and 75 years *co-morbidity*: pain symptoms (subjective assessment by the participant) *IG*: 48.62 (11.27), 78.9% *CG*: 47.68 (12.47), 76.1%	RCT: TAU + DiGA, *n* = 71 TAU (WLC), *n* = 71	severity of depressive symptoms (BDI-II)	IG: 32,4% CG: 15,5%	ITT (EM) Δ = N/A, *p* = 0.01 ηp² = 0.043, 95% CI = N/A
Bücker et al. (2018)^27^	Deprexis (8 weeks)	*depressive symptoms*: subjective feelings of sadness and desperation *age*: between 18 and 65 years *co-morbidity*: self-reported problem or pathological gambling with slot machines IG: 34.42 (10.74), 23.94% CG: 37.04 (9.53), 23.19%	RCT: IG, *n* = 74 WLC, *n* = 71	severity of depressive symptoms (PHQ-9)	IG: 56.3% CG: 39.1%	ITT (EM) Δ = N/A, *p* = 0.001 ηp² = 0.125, 95% CI = N/A
Moritz et al. (2012)^28^	Deprexis (8 weeks)	*depressive symptoms*: participants were invited in online platforms for people with depression to exchange opinions and advice *age*: between 18 and 65 years IG: 38.00 (10.76), 77.1% CG: 39.13 (15.82), 80.0%	RCT: IG, *n* = 105 WLC, *n* = 105	depression severity (BDI)	IG: 23,81%, CG: 14,29%	ITT (MI) Δ = N/A, *p* = 0.03 d = 0.36, 95% CI = N/A
Schröder et al. (2014)^29^	Deprexis (9 weeks)	*depressive symptoms*: subjective complaints of depressive symptoms *co-morbidity*: self-reported epilepsy diagnosis IG: 35.03 (9.99), 84.21% CG: 40.03 (11.85), 67.5%	RCT: IG, *n* = 38 WLC, *n* = 40	depression severity (BDI)	IG: 37% WLC: 20%	ITT (LOCF) Δ = N/A, *p* = 0.04 d = 0.29, 95% CI = N/A
Meyer et al. (2009)^30^	Deprexis (9 weeks) FU: 6 months	*depressive symptoms* *age*: adults *extra*: completed at least half of the baseline depression questionnaire IG: 34.58 (11.53), 77% CG: 35.47 (11.98), 71%	RCT: DiGA + TAU, *n* = 320 TAU, *n* = 76	depression severity (BDI)	*Post-intervention*: IG: 50%, CG: 25% *Follow-up*: IG: 73%, CG: 81%	ITT (LOCF) *Post-intervention*: Δ = −3.46, *p* = 0.002 d = 0.30, 95% CI = 0.05, 0.55 *Follow-up*: N/A
Fischer et al. (2015)^31^	Deprexis (9 weeks) FU: 6 months	*depressive symptoms*: self- reported depressive symptoms *age*: between 18 and 65 years, *extra*: willingness to self- administrate iCBT sessions for 9 weeks *co-morbidity*: diagnosis of multiple sclerosis *IG*: 45.36 (12.64), 76.00% *CG*: 45.20 (10.56), 80.00%	RCT: IG, *n* = 35 WLC, *n* = 36	severity of depressive symptoms (BDI)	*Post-intervention*: IG: 22% CG: 20% *Follow-up*: IG: 0% CG: N/A	ITT (LOCF) *Post-intervention*: Δ = −4.02, 95% CI = −7.26, −0.79, *p* < 0.01 d = −0.53, 95% CI = N/A *Follow-up*: N/A
**Kalde et al. (2025)^32^	Elona therapy (12 weeks)	*depressive symptoms*: diagnosed with ICD-10 diagnosis (F32, F33, F34.1) *age*: between 18 and 65 years *extra*: sufficient German language skills, smartphone possession, signed and dated consent, questionnaires within 14 days *IG*: 37.52 (10.90), 75% *CG*: 38.18 (10.86), 73%	RCT: bCBT (TAU + DiGA), *n* = 144 TAU, *n* = 139	depressive symptoms (PHQ-9)	IG: 4.17%, CG: 11.51%	ITT (JTR) Δ = N/A, *p* = 0.001 d = 0.62, 95% CI = 0.37, 0.86
Beiwinkel et al. (2017)^33^	Novego (Help-ID) (12 weeks) FU: 6 months	*depressive symptoms*: previous diagnosis of depression (ICD F32.0, F32.1, F33.0, F33.1, F34.1), previous sickness absence due to depression, and current sickness absence *age*: adults *IG*: 47.01 (10.36), 66.0% *CG*: 48.66 (11.59), 71.3%	RCT: TAU + DiGA, *n* = 100 TAU (WLC -access to unguided Web-based psycho-education), *n* = 80	depressive symptoms (PHQ-9)	*Post-intervention*: IG: 56% CG: 45% *Follow-up*: IG: 69% CG: 66.25%	ITT (MI) *Post-intervention*: Δ = −1.25, *p* < 0.001 d = 0.55, 95% CI = 0.25, 0.85 *Follow-up*: N/A
Klein et al. (2016)^34^	Deprexis (12 weeks) FU: 6 months	*depressive symptoms*: presence of self-reported mild to moderate depressive symptoms (PHQ-9 score: 5–14) *age*: between 18 and 65 years *IG*: 42.8 (11.0), 68.8% *CG*: 42.9 (11.0), 68.5%	RCT: TAU + DiGA, *n* = 509 TAU, *n* = 504	depression severity (PHQ-9)	*Post-intervention*: IG: 22.4%, CG: 20.8% *Follow-up*: IG: 25.74% CG: 25.40%	ITT (MI) *Post-intervention*: Δ = −1.61, *p* < 0.001 d = −0.39, 95% CI = 0.13, 0.64 *Follow-up*: Δ=−1.38, p = N/A d = −0.32, 95% CI = 0.06, 0.59
Zwerenz et al. (2017)^35^ Zwerenz et al. (2019)^25^	Deprexis (12 weeks) FU: 6 months	*depressive symptoms*: BDI-II > 13, clinical diagnosis of depression *age*: between 18 to 65 years *IG*: 47.36 (10.38), 64.3% *CG*: 48.61 (9.16), 57%	RCT: TAU (inpatient psychotherapy) + DiGA, *n* = 115 TAU, *n* = 114	depressive symptoms (BDI-II)	*Post-intervention*: IG: 26.09% CG: 25.44% *Follow-up*: IG:24.4%, CG:36.0%	ITT (MI) *Post-intervention*: Δ = −4.65, *p* = 0.001 d = −0.44, 95% CI = N/A *Follow-up*: Δ = −6.23, *p* < 0.001 d = 0.58, 95% CI = N/A
Moritz et al. (2016)^36^	Novego (HelpID) (12 weeks)	*depressive symptoms*: present subjective depressive symptoms and willingness to undergo treatment for these symptoms (no formal cut-off) *age*: between 18 and 65 years *co-morbidity*: diagnosis of schizophrenia (verified by telephone interview) *IG*: 38.19 (11.78), 45.17% *CG*: 43.41 (8.42), 62.97%	RCT: DiGA, *n* = 31 WLC, *n* =- 27	depressive symptoms (PHQ-9)	IG: 19,35%, CG: 11,12%	ITT (EM) Δ = N/A, *p* = 0.001 ηp² = 0.179, 95% CI = N/A
Meyer et al. (2014)^37^	Deprexis (12 weeks) FU: 6 months	*depressive symptoms*: PHQ-9 score of > 14 in the EVIDENT screening *age*: between 18 and 65 years *extra*: written informed consent *IG*: 44 (11.02), 74.4% *CG*: 40 (11.48), 75.3%	RCT: CAU + DiGA, *n* = 78 CAU (WLC), *n* = 85	depressive symptom severity (PHQ-9)	*Post-intervention*: IG: 22%, CG: 14% *Follow-up*: IG: 27%, CG: 18%	ITT (MLE) *Post-intervention*: Δ = −3.56, *p* < 0.01 d = 0.57, 95% CI = 0.22, 0.92 *Follow-up*: Δ = −2.11, *p* < 0.01 D = 0.33, 95% CI = −0.03, 0.69
Berger et al. (2011)^38^	Deprexis (10 weeks) FU: 6 months	*depressive symptoms*: score of > 13 on the BDI-II, score < 2 on the suicide item of the BDI *age*: adults *extra*: not participating in any other psychological treatment for the duration of the study, and if on prescribed medication for depression/anxiety, dosage had to be constant for 1 month before the start of the treatment and the participant had to agree to keep the dosage constant throughout the study *Guided IG*: 38.2 (15.1), 68.0% *Unguided IG*: 38.6 (14.2), 72.0% *CG*: 39.6 (13.2), 69.2%	RCT: Guided IG, *n* = 25 Unguided IG, *n* = 25 WLC, *n* = 26	depression severity (BDI-II)	*Post-intervention*: Guided IG: 0%, Unguided IG: 12% CG: 15.39%, *Follow-up*: Guided IG: 20%, Unguided IG: 24%, CG: 23.08%	ITT (LOCF) *Post-intervention*: guided vs. WLC Δ = −11.2, *p* < 0.001 d = 1.14, 95% CI = N/A unguided vs. WLC Δ = −7.7, *p* = 0.02 d = 0.66, 95% CI = N/A *Follow-up*: unguided vs. WLC Δ = −1.68, p = N/A d = 0.26, 95% CI = N/A
Richter et al. (2023)^39^	Deprexis (12 weeks)	*depressive symptoms*: admitted in hospital for treatment of MDD (inpatient psychotherapy), Mini-Mental-State-Test > 24 *age*: between 18 and 65 years *IG*: 49.0 (12.24), 46.9% *CG*: 42.5 (13.67), 46.9%	RCT: DiGA + TAU, *n* = 36 TAU, *n* = 33	depression severity (BDI-II)	IG: 37.5% CG: 46.9%	ITT (MI) Δ = −8.36, *p* = 0.03 d = −0.73, 95% CI = −1.4, 0.06
Lopes et al. (2023)^40^	Deprexis (12 weeks)	*depressive symptoms*: clinically relevant depressive symptoms (PHQ-9: ≥ 10), diagnosed with a depressive disorder or dysthymia following the definitions of DSM-5 *age*: Adults *IG*: 35.82 (10.76), 83% *CG*: 37.06 (12.27), 84%	RCT: TAU + DiGA, *n* = 94 TAU (WLC) n = 95	depressive symptoms (PHQ-9)	IG: 41,8% CG: 31%	ITT (LOCF) Δ=−6.77, *p* < 0.001 d = 0.80, 95% CI = 0.51, 1.10
Beevers et al. (2017)^41^	Deprexis (8 weeks)	*depressive symptoms*: presence of moderate levels of depression or greater (QIDS score ≥ 10) at time of eligibility screening *age*: between 18 and 55 years *extra*: treatment stability, willingness to provide saliva for DNA research *IG*: 31.2 (10.7), 25.60% *CG*: 34.1 (12.4), 22.0%	RCT: IG, *n* = 285 WLC, *n* = 91	depressive symptoms (QIDS-SR)	IG: 21,8% CG: 32,97%	ITT (MI) Δ = −4.09, *p* < 0.001 d = −0.80, 95% CI = 0.56, 1.04
Krämer et al. (2022)^42^	selfapys for depression (12 weeks) FU: 6 months	*depressive symptoms*: BDI-II score ≥ 13, diagnosis of a major depressive disorder or dysthymia based on the MINI, in accordance with the International Statistical Classification of Diseases tenth revision (ICD-10: F32, F33, F34) *age*: between 18 and 65 years *extra*: willingness to provide electronic data *IG guided*: 38 (10.7), 83.4% *IG unguided*: 37 (10.8), 84.0% *CG*: 36 (11.9), 81.0%	RCT: Guided IG, *n* = 151 Unguided IG, *n* = 30 WLC, *n* = 100	depressive symptoms (BDI-II)	*Post-intervention*: IG guided: 12.6%, IG unguided: 22.6%, CG: 47.0% *Follow-up*: IG guided: 57.62% IG unguided: 8.67% CG: 71%	ITT (MI) *Post-intervention*: guided vs. WLC Δ = −13.65, *p* < 0.001 d = 1.63, 95% CI = 1.37, 1.93 unguided vs. WLC Δ = −11.77, *p* < 0.001 d = 1.47, 95% CI = 1.22, 1.73 *Follow-up*: N/A
Berger et al. (2017)^43^	Velibra (9 weeks) FU: 6 months	*anxiety symptoms*: confirmed SAD, PDA and/or GAD diagnosis, *age*: ≥ 18 years *extra*: if taking prescribed medication for depression/anxiety, the dosage had to be constant for at least 1 month before the start of the treatment *IG*: 42.1 (12.2), 69.0% *CG*: 41.8 (12.2), 72.0%	RCT: CAU + DiGA, *n* = 70 CAU (active WLC), *n* = 69	anxiety and depression symptom severity (DASS-21)	*Post-intervention*: IG: 19%, CG: t1 9% *Follow-up*: IG: 37% CG: N/A	ITT *Post-intervention*: Δ = −11.8, *p* < 0.01 d = 0.47, 95% CI = 0.13, 0.81 *Follow-up*: N/A
Rubel et al. (2024)^44^	selfapys for GAD (12 weeks)	*anxiety symptoms*: currently met criteria for a diagnosis of GAD (DSM-5300.02) *age*: between 18 and 65 years *IG*: 33.2 (10.5), 88.46% *CG*: 37.2 (12.4), 75.64%	RCT: TAU + DiGA, *n* = 78 TAU (WLC), *n* = 78	generalized anxiety symptoms (GAD-7)	IG: 12.8% CG: 26.93%	ITT (MI) Δ = −2.87, *p* < 0.001 d = 0.88, 95% CI = 0.50, 1.26

All information was extracted from the original study publications. If no change score (Δ) was reported by the study autors, “N/A” is used. Results at post-intervention refer to the primary outcome listed in Column 5.

TAU=Treatment as usual; BDI=Beck Depression Inventory; PHQ-9 = Patient Health Questionnaire (9 items); IG=Intervention group; CG=Control group; ITT=Intention-to-treat analysis; LOCF=Last observation carried forward; MI=multiple imputation; MDD=major depressive disorder; DSM-5 = Diagnostic and Statistical Manual of Mental Disorders, Fifth Edition; ICD-10=.

International Statistical Classification of Diseases, tenth revision; EM=expectation-maximization; MLE=Maximum likelihood estimation; JTR= jump to reference; bCBT=blended cognitive behavioral therapy; QIDS=Quick Inventory of Depression Symptoms; MINI=Mini International Neuropsychiatric Interview; SAD=Social anxiety disorder; PDA=Panic disorder with or without agoraphobia.

*Zwerenz et al. (2019) reports follow-up data of the RCT of Zwerenz et al. (2017).

**pre-print.

### Quality appraisal – risk of bias assessment

Two independent reviewers (SF and AS) assessed the risk of bias for all RCTs for assignment to intervention (the ‘intention-to-treat’ effect) using the Cochrane RoB2 tool^45^. Inter-rater agreement was substantial, with a Cohen’s k of 0.53, indicating a moderate level of consistency in ratings. Discrepancies were resolved through discussion with the third reviewer (AK).

**Fig. 2:**
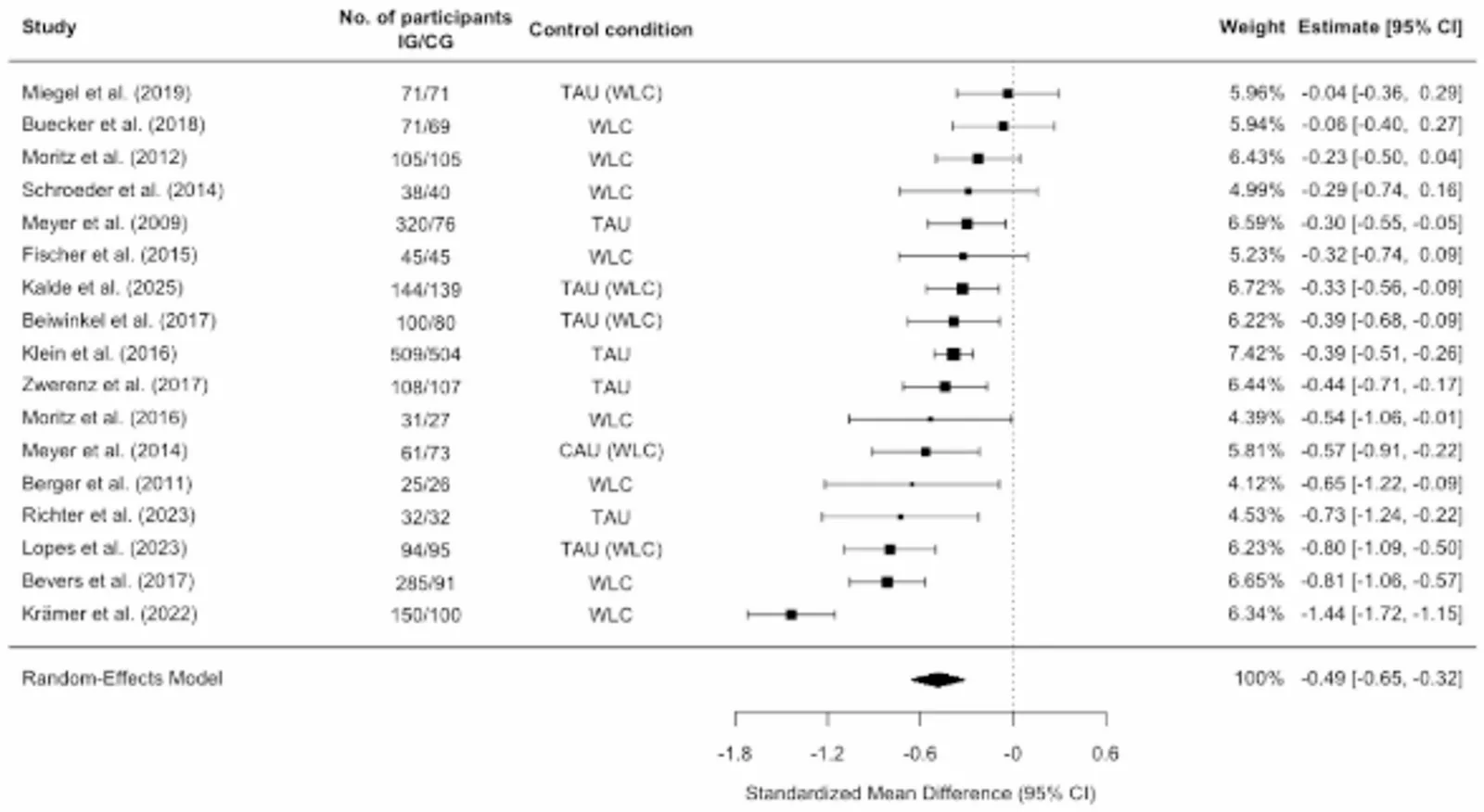
Effect of DiGA on depression. Forest plot of the effects of interventions on depression severity (SMD, 95% CI) at post-intervention including 17 RCTs (n = 3,869 total participants). Results of a two-sided random-effects meta-analysis. This figure displays the main analysis of the intervention effect on the observed outcome across studies using a random-effects model. The analysis summarizes post-intervention data comparing intervention groups (IG) and control groups (CG) across 17 studies. Effect sizes are presented as standardized mean differences (SMD) with corresponding 95% confidence intervals (CI). The horizontal lines represent the 95% CI for each individual study, while the black squares denote the point estimates; square size reflects the relative study weight in the meta-analysis. The pooled effect size is shown as a black diamond at the bottom, with its width indicating the 95% CI. Statistical significance is inferred based on 95% confidence intervals; two-sided p-values < 0.05 are assumed when the CI does not cross zero.

**Fig. 3:**
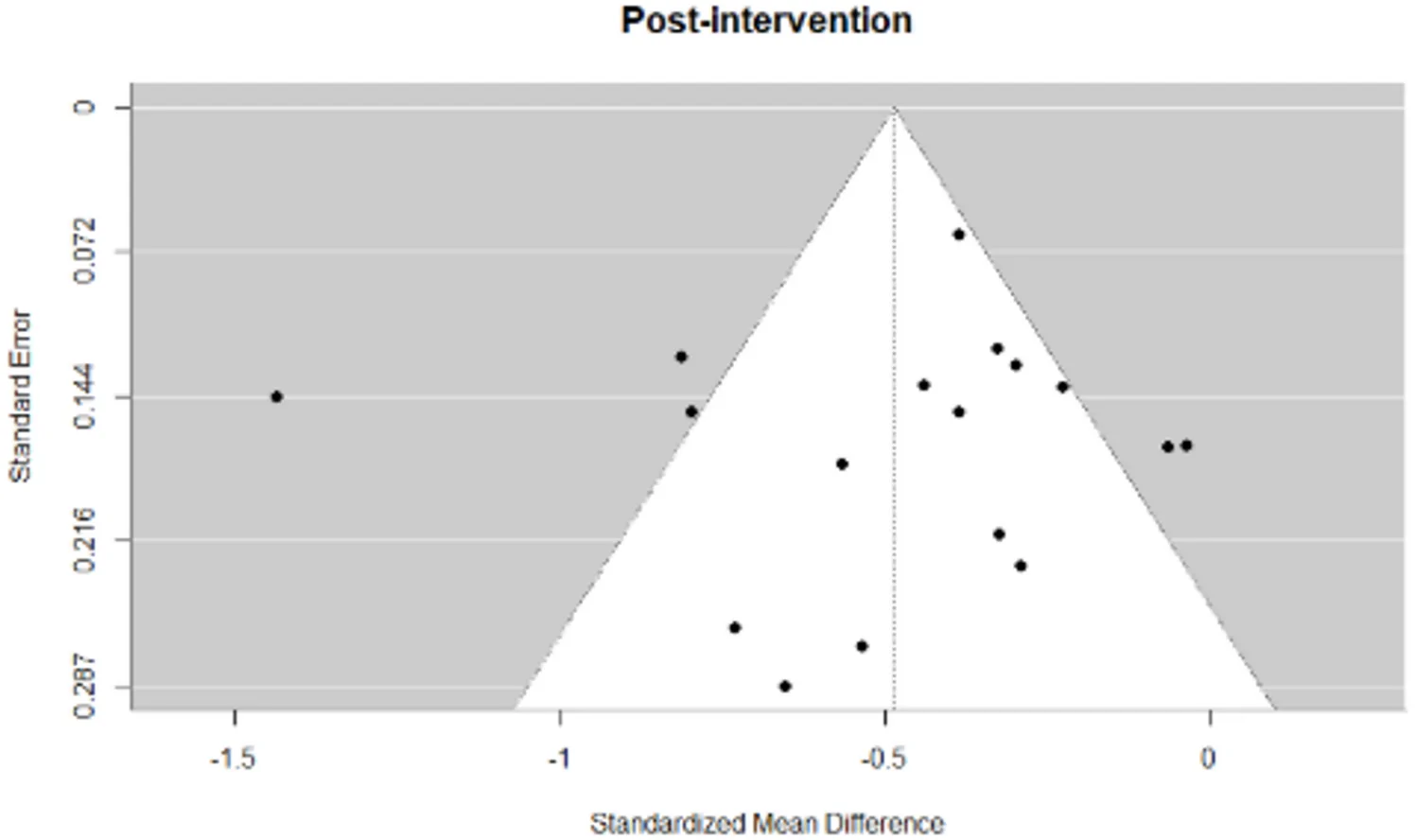
Funnel plot of DiGA treating Depression. The observed outcome (SMD on X-axis) of each study with respect to its standard error (Y-axis) was plotted to evaluate publication bias, and the funnel plot was visually inspected for asymmetry.

Overall, the risk of bias was judged as low in domain 1 (Randomization process) and domain 2 (Deviations from the intended interventions) in the majority of studies, reflecting appropriate allocation concealment procedures and adherence to intervention protocols. In contrast, domain 3 (Missing outcome data) and domain 4 (Measurement of the outcome) were associated with high risk of bias across nearly all studies. The primary reasons were high attrition rates and the use of patient-reported outcome measures (PROMs), such as the PHQ-9 for depression severity. These outcomes were assessed by the participants themselves, who were unblinded to treatment allocation, introducing a substantial risk of detection bias. Consequently, all included trials were rated as having an overall high risk of bias, primarily due to systematic concerns in domains 3 and 4. Domain 5 (Selection of the reported result) was judged at high risk in four studies, due to inconsistencies between protocols and reported outcomes. In contrast, one study provided both a pre-specified protocol and a comprehensive statistical analysis plan (SAP)^44^, which substantially enhanced transparency and facilitated a more reliable assessment of the risk of selective reporting. Full risk of bias judgements for each domain and study are provided in Supplementary Material S1 Fig. 2. A graphical summary of domain-specific risk distributions is presented in Supplementary Material S1 Fig. 3.

### Meta-analytical results

The meta-analytical results are presented separately for depression and GAD.

#### Depression – post-intervention

A random effects meta-analysis of 17 RCTs evaluating DiGAs for depressive symptoms was conducted, synthesizing data from a total of 3,869 participants (IG 2,189/CG 1,680). Results indicate a statistically significant effect in favor of the intervention (SMD = −0.49; 95% CI: −0.65, −0.32; *p* < 0.0001), indicating that DiGAs were more effective than control conditions in reducing depressive symptoms at post-intervention. There was substantial heterogeneity among studies (Q(16) = 81.04; *p* < 0.0001; I^2^ = 81.79%; 95% CI: 64.82, 91.84; 95% PI: −1.11, 0.14), indicating considerable variability in effect sizes. The between-study variance (τ²) was estimated at 0.10. A forest plot displaying individual and pooled effect sizes is presented in Fig. 2. Deprexis dominated the available evidence, thus the pooled estimates primarily reflect the evidence base for this application rather than an equal representation of all currently listed DiGAs for depression.

Potential publication bias was evaluated using multiple complementary approaches. Visual inspection of the funnel plot (Fig. 3) suggested slight asymmetry. However, Egger’s regression test did not indicate significant asymmetry (*p* = 0.86), suggesting that small-study effects unlikely have systematically influenced the overall effect estimate. To further assess potential bias, a trim-and-fill analysis was conducted. This procedure estimated that no studies are missing. Additionally, the Rosenthal fail-safe N was calculated to quantify the robustness of the findings. A total of 1,063 null-effect studies would be required to reduce the overall effect to non-significance, further supporting the stability of the observed result. Overall publication bias is unlikely to pose a significant threat to the validity of the present meta-analytic conclusions.

### Sensitivity analyses

To assess the robustness of the main result, a sensitivity analysis was performed excluding one influential study. The effect remained significant in favor of the intervention (SMD= −0.42; *p* < 0.0001), though heterogeneity was reduced (Q(15) = 33.28; *p* = 0.004; I^2^ = 58.93%), residual heterogeneity remained. The 95% prediction interval (−0.78, −0.06) indicates robust and consistent beneficial effects. The study excluded in this sensitivity analysis was identified through a preceding influence analysis, which was conducted to detect studies with a disproportionate impact on the pooled estimate. One study was identified as influential, reporting a notably large effect size (SMD= −1.44)^42^. This study was the only trial evaluating the app *Selfapys Online Kurs bei Depression*, which may account for its divergence from the rest of the sample.

Five additional sensitivity analyses were conducted to evaluate the robustness of the main effect (Table 2). (I) Risk of bias: A sensitivity analysis excluding studies rated as ‘high risk’ in three or more RoB 2 domains showed consistent results, which indicates that the main findings were not driven by studies with elevated risk of bias. (II) Population severity: To assess the impact of baseline symptom severity, studies that explicitly recruited participants with severe depression at baseline were removed. The effect size remained stable, suggesting generalizability across clinical severity levels. (III) Intervention guidance: Another analysis excluded studies using guided DiGA interventions. Results were consistent, supporting the efficacy of both guided and unguided formats. (IV) Per-protocol analysis: For studies reporting per-protocol data, an additional analysis was performed. The pooled effect was larger (SMD = −0.67), although the estimate was less precise (SE = 0.12; 95& CI: −0.92, −0.43). (V) Alternative random-effects estimators: Sidik-Jonkmann, Paule-Mandel, Hartung-Knapp-Sidik-Jonkmann approaches were used to assess the robustness of the pooled effect estimates against different assumptions about between-study variance. Across all sensitivity analyses, the direction and magnitude of the effect remained consistent, and all models retained statistical significance (*p* < 0.05), although the 95% prediction interval mostly included zero, indicating possible variation.

**Table 2:** Sensitivity analysis.

Sensitivity	*N* of studies (*n*_IG_/*n*_CG_)	SMD	SE	*P*	95% CI	Q(df), *p*	I^2^	τ²	95% PI
Without Outliers	16 (2,039/1,580)	−0.42	0.06	< 0.0001	(−0.53, −0.29)	33.28(15), 0.0043	58.93%	0.03	(−0.78, −0.06)
Without High Risk of bias	14 (1,733/1,440)	−0.50	0.10	< 0.0001	(−0.69, −0.31)	67.19(13), < 0.0001	82.22%	0.31	(−1.14, 0.15)
Without Severe depression	16 (2,128/1,607)	−0.48	0.10	< 0.0001	(−0.6, −0.30)	80.72(15), < 0.0001	83.10%	0.10	(−1.13, 0.17)
Per-protocol sample	13 (1,224/1,003)	−0.67	0.12	< 0.0001	(−0.92, −0.43)	66.53(12), < 0.0001	83.72%	0.15	(−1.48, 0.14)
Without Guided Intervention	16 (2,157/1,648)	−0.47	0.09	< 0.0001	(−0.65, −0.30)	79.99(15), < 0.0001	82.95%	0.10	(−1.11, 0.16)
Sidik- Jonkmann	17 (2,189/1,680)	−0.49	0.08	< 0.0001	(−0.65, −0.32)	81.04(16), < 0.0001	81.36%	0.09	(−1.10, 0.13)
Paule-Mandel	17 (2,189/1,680)	−0.49	0.08	< 0.0001	(−0.64, −0.32)	81.04(16), < 0.0001	80.80%	0.09	(−1.09, 0.12)
Hartung-Knapp-Sidik-Jonkmann	17 (2,189/1,680)	−0.49	0.08	< 0.0001	(−0.66, −0.31)	81.04(16), < 0.0001	81.36%	0.09	(−1.15, 0.18)

### Moderator/subgroup analyses

Categorical moderator analyses were conducted to examine whether intervention effects varied systematically across study characteristics (Supplementary Material S3 Table S2). The type of DiGA was a significant moderator of treatment efficacy (QM(3) = 39.98; *p* < 0.0001), indicating substantial between-group differences across different digital applications. However, this needs to be interpreted with caution, as *Deprexis* was evaluated in a significantly higher number of studies than other DiGAs, potentially amplifying its influence on the moderation results.

 Additional moderator analyses were performed for treatment setting, comorbidity status, and country. None of the overall moderator tests were statistically significant. Although some regression coefficients were statistically significant relative to the reference category, these findings should be interpreted with caution because the overall moderator tests were not significant and several subgroup analyses were based on a small number of studies.

To investigate the influence of continuous variables on intervention effects, continuous moderator analyses were conducted (Supplementary Material S3 Table S3). None of the variables (intervention duration (in weeks), mean age of participants, percentage of female participants, and total sample size) significantly predicted the magnitude of the treatment effect (*p* > 0.05). Nevertheless, the percentage of female participants accounted for 4.79% of the between-study variance. However, residual heterogeneity remained high and statistically significant, suggesting that additional, unmeasured factors contribute to variability in effect sizes across studies.

#### Depression - follow-up

Follow-up data were reported in nine studies. However, only four studies could be included in the quantitative synthesis. The remaining studies either reported follow-up data only for the per-protocol/completer population^30,42^ or only for the intervention group^31,43^ or only for within-group effects^33^. Follow-up data were only available for studies examining *Deprexis*, which had used comparable intervention duration (10–12 weeks) and follow-up periods (6 months) in a German-speaking population. Given this clinical and methodological similarity among these studies, a fixed-effects model was applied.

The meta-analysis yielded a statistically significant pooled effect size in favor of the intervention group (SMD = −0.35; *p* < 0.0001; Q(3) = 3.41; *p* = 0.33, Supplementary Material S1 Fig. 4). Statistical heterogeneity was low (I^2^ = 12.08%), supporting the assumption of homogeneity. An influence analysis revealed that two studies^25,34^ exerted disproportionate influence on the overall estimate.

Egger’s regression test was not statistically significant (*p* = 0.82), but the assessment of potential publication bias via funnel plot inspection (Supplementary Material S1 Fig. 5) suggested existing asymmetry. Fail-safe N analyses indicated a moderate vulnerability to bias: Rosenthal’s *N* = 46. These findings suggest that *Deprexis* may have sustained effects on depressive symptoms at 6-month follow-up, although the evidence base remains limited and sensitive to individual study effects.

### Generalized anxiety disorder

Only two RCTs targeting GAD met the inclusion criteria for meta-analysis^43,44^. Individual study effects are presented in Table 1. Although both studies favored the intervention, the estimated treatment effects differed markedly in magnitude, resulting in considerable between-study heterogeneity. The pooled effect was neither statistically significant nor precise (Table 3), primarily due to substantial heterogeneity across studies (I^2^ = 98.84%; Q = 85.99; *p* < 0.0001; τ² = 4.66; 95% PI = −7.50, 2.89). Given that the meta-analysis was based on only two trials, the pooled effect should be interpreted with caution and does not permit robust conclusions regarding the efficacy of DiGAs for GAD.

**Table 3:** Individual study effects and random-effects Model for studies evaluating DiGA for GAD.

Study (Year)	*N*_IG_	*N*_CG_	DiGA	Effect Size	95% CI	Random-Effects Model
Rubel et al. (2014)	78	78	Selfapy for GAD	−0.78	(−1.11, −0.46)	− 2.31 95% CI: (−5.32, 0.70) (*p* = 0.1330)
Berger et al. (2017)	70	69	velibra	−3.85	(−4.41, −0.29)	

### GRADE

The certainty of evidence was evaluated using the GRADE approach for all three outcomes: depression post-intervention; depression follow-up; and GAD post-intervention, based on pooled effect estimates from the ITT-populations^46^. Overall, the quality of evidence was low to very low (Table 4). Primary reasons for downgrading were: risk of bias, particularly due to lack of blinding, as participants also served as outcome assessors in most studies and high attrition rates, which introduced additional risks of bias related to incomplete outcome data, substantial statistical heterogeneity across studies, leading to downgrading for inconsistency. For GAD, wide confidence intervals crossing the line of no effect, further reduced certainty due to imprecision.

These ratings highlight considerable limitations in the current evidence base – particularly with respect to methodological quality, inconsistency, and the robustness of effect estimates. As such, the results should be interpreted with caution.

**Table 4:** GRADE Summary of findings.

Outcome	Anticipated absolute effects (95% CI)	№ of participants (studies)	Quality of the evidence (GRADE)	Comments
Depression (post-intervention)	−0.49 (−0.65, −0.32)*	3,869 (17)	⨁◯◯◯ ^a, b^ VERY LOW	A SMD of − 0.49 represents a moderate effect in favor of the intervention group (with negative values indicating a reduction in symptoms).
Depression (follow-up)	−0.35 (−0.46, −0.29)**	1,407 (4)	⨁⨁◯◯ ^a^ LOW	A SMD of −0.35 represents a moderate effect in favor of the intervention group (with negative values indicating a reduction in symptoms).
Generalized Anxiety Disorder (post-intervention)	−2.31 (−5.32, 0.70)*	295 (2)	⨁◯◯◯ ^a, b,c, d^ VERY LOW	A SMD of − 2.31 indicates a large effect in favor of the intervention group; however, the result is not statistically significant and shows considerable imprecision.

* Based on pooled effect estimates using a random-effects model (REML) of the intention-to-treat sample.

** Based on pooled effect estimates using a fixed-effect model of the intention-to-treat sample.

^a^ We downgraded quality due to missing blinding of participants and outcome assessors and high drop-out rates.

^b^ We downgraded for inconsistency due to studies having high heterogeneity.

^c^ We downgraded for imprecision as the upper and lower limits of the confidence intervals include both potential for harm and potential for benefit.

^d^ We downgraded for publication bias as only 2 studies were included.

GRADE Working Group grades of evidence.

High quality: We are very confident that the true effect lies close to that of the estimate of the effect.

Moderate quality: We are moderately confident in the effect estimate: The true effect is likely to be close to the estimate of the effect, but there is a possibility that it is substantially different.

Low quality: Our confidence in the effect estimate is limited: The true effect may be substantially different from the estimate of the effect.

Very low quality: We have very little confidence in the effect estimate: The true effect is likely to be substantially different from the estimate of effect.

## Discussion

This meta-analysis showed a statistically significant symptom reduction by DiGAs for depression (SMD = −0.49; 95% CI: −0.65, −0.32; *p* < 0.0001; *n* = 17), especially for *Deprexis* (SMD = −0.46; 95% CI: −0.60, −0.32; *p* < 0.0001; 95% PI: −0.85, −0.07; *n* = 12). Effects of DiGAs for GAD are imprecise according to *GRADE* due to a lack of RCTs (SMD= −2.31; 95% CI: −5.32, 0.70, *n* = 2). The effects of DiGAs for depression were consistent across sensitivity analyses and comparable in magnitude to those reported for traditional face-to-face psychotherapies^47^. However, this does not imply equivalence, as no direct comparative trials exist. Moreover, in the study analysed approximately 20% of participants received concurrent psychotherapy and about 30% were taking psychotropic medication, including antidepressant, at baseline. The observed effect sizes may therefore at least partially reflect combined rather than isolated DiGA treatment effects.

From a clinical point of view, a striking limitation of the current evidence is the age distribution of participants. Despite the high prevalence of depression in older adults, the weighted pooled mean age of participants in the intervention groups was 38.7 years (SD = 12.1). Although trials did not explicitly exclude older adults, data on individuals over 70 years, an especially relevant group, are absent. Non-German-speaking individuals and those with lower educational attainment were also underrepresented, suggesting selection bias toward resource-strong, health-literate users. This highlights important barriers to digital health equity and challenges the assumption of universal accessibility.

An important limitation concerns the unequal distribution of evidence across DiGAs. Twelve of the nineteen included RCTs evaluated *Deprexis*, whereas some permanently listed DiGAs were represented by only a single study or by no eligible RCT at all. Moreover, some *Deprexis*-trials were conducted before the introduction of the German DiGA fast-track process in 2020. Although *Deprexis* now fulfils the criteria for permanent DiGA listing these earlier trials were performed within a different regulatory context. Consequently, the pooled effect estimates reflect the currently available evidence base rather than providing an equally representative estimate for each included DiGA. The evidence base for GAD was particularly limited, with only two eligible RCTs available. GAD was retained in accordance with the registered and published protocol^21,48^. However, given the very small number of studies, the pooled estimate should be interpreted with considerable caution and does not permit robust conclusions regarding the effectiveness of DiGAs for GAD. Moreover, some included trials were conducted outside of Germany. By including all available RCTs, this review provides the most comprehensive synthesis of the evidence for each DiGA irrespective of country. Nevertheless, differences in healthcare systems, implementation pathways, and routine care may limit the direct transferability of these findings to the German healthcare setting.

Follow-up assessments were short in duration (6 months), and while some studies demonstrated effects, evidence on long-term sustainability remains scarce. A further limitation arises from restricting the review to permanently listed DiGAs. These interventions represent a regulatory-selected group that has already demonstrated a positive healthcare effect, while non-listed, rejected, or delisted applications were not considered. This may result in more favourable pooled effect estimates than would be expected for digital therapeutics more broadly and limits the generalizability of the findings beyond the German DiGA framework. However, this restriction was consistent with the aim of evaluating prescription-eligible interventions currently available within statutory healthcare.

The studies evaluated DiGAs in a range of control conditions, commonly treatment-as-usual, care-as-usual and/or wait-list control. However, these control conditions lacked detailed characterization of the intensity and quality of treatment conditions, especially in outpatient settings. Consequently, it remained unclear whether participants received structured psychological or pharmacological treatment, routine primary care or no active treatment at all. As highlighted by Munder et al. (2021), the intensity of TAU can significantly moderate the effect sizes observed in both face-to-face and internet-based psychotherapy^49^. Therefore, differences in control group quality across studies may have influenced the observed treatment effects and should be considered when interpreting the results. Another aspect that warrants consideration is the treatment setting in which DiGAs are implemented. To provide a comprehensive overview of the available evidence, studies were included conducted in both inpatient and outpatient setting and examined in a sensitivity analyses. However, these broad categories are unlikely to capture the complexity of routine care. Treatment outcomes may also depend on the healthcare professional initiating the prescription (e.g. psychotherapists or primary care physicians), the degree of therapeutic support provided alongside the DiGA or its integration into existing pathways. These implementation-related factors cannot be adequately addressed within this analysis and should be investigated in future real-world effectiveness studies.

From a methodological point of view, all studies used reliable and valid PROMs^50^. Formally, this leads to a high risk of bias; however, PROMs remain the international standard in depression and GAD research. Lack of blinding is inevitable but may lead to systematic overestimation. Placebo or sham controls minimize expectancy effects, but studies with true placebo or sham conditions are conceptually challenging, if not impossible, in psychological and digital interventions^51,52^. Unlike pharmacological studies, users actively engage with content, meaning, and expectations, which are inseparable from the therapeutic mechanism itself. Even in sham-controlled designs, expectation effects remain present, and controlling for them may obscure the real-world impact of interventions. This interpretation is further supported by experimental evidence demonstrating that expectancy induction alone may influence outcomes in smartphone-based mental health interventions^53^. Another methodological limitation is the high dropout rates observed, suggesting that reported effects may primarily reflect the adherence of more motivated or digitally literate users. This phenomenon is particularly relevant in depression, where core symptoms such as loss of energy and diminished interest may reduce regular engagement with digital interventions. Furthermore, the manufacturers involvement deserves consideration. Although the extent of involvement varied across studies, no included trial was entirely free from manufacturer involvement. Such collaborations are common in the evaluation of regulated digital health technologies and do not necessarily compromise study validity, they may introduce potential sources of bias in study design, conduct, analysis or reporting.

In this meta-analysis, there was substantial heterogeneity of intervention formats, settings, study populations, control conditions and outcome measures, which complicates interpretation despite the use of random-effects models. Although sensitivity analyses confirmed the robustness of the main findings, publication bias cannot be ruled out. The reliance on a random-effects meta-analytic model implies an assumption of variation across true effects, but some critique this approach for overweighting small studies or inflating confidence in unstable estimates^54^. Moreover, this meta-analysis did not restrict for a specific ITT-approach; instead, studies with divergent ITT-methods were included. This methodological variability may have influenced individual effect sizes and limits comparability across studies.

This structured evaluation of DiGAs could potentially guide approaches in different therapeutic areas. Given its prominence in the DiGA directory and the comparatively robust evidence base, depression serves as a useful case for such an evaluation. Overall, the currently available evidence indicates that prescription-eligible DiGAs – predominantly *Deprexis* – can reduce depressive symptoms. DiGAs for depression show promising efficacy, comparable in magnitude to traditional psychotherapies. However, the methodological limitations, heterogeneity, and real-world barriers identified call for cautious interpretation and underscore the urgent need for high-quality, pragmatic trials that assess both efficacy and effectiveness across diverse populations and contexts. As DiGAs are primarily self-guided, their effectiveness may strongly depend on user motivation and engagement, which vary widely in routine care, especially in patients with depression. In addition, usability, digital literacy, and accessibility are rarely measured but may substantially moderate real-world effectiveness. There is a need for future studies that address both clinical efficacy and the content-related aspects of DiGAs, such as the therapeutic approaches used (e.g., CBT modules, psychoeducation, behavioural activation) and their relevance for different subgroups of patients with depression. As the number of approved DiGAs and published evaluations continues to grow, regular updates of the evidence base will be essential to provide an up-to-date assessment of their efficacy and to inform clinical practice and healthcare decision-making.

## Methods

This systematic review and meta-analysis was conducted in accordance with the PRISMA 2020 guidelines (Supplementary Material S3 Table S4)^55^. The review protocol was registered (PROSPERO: CRD42024557629)^48^ and reported following the PRISMA-P checklist, with the completed checklist provided in the published protocol^21^.

### Search strategy: DiGA

DiGAs were identified via the official BfArM DiGA directory, following the systematic app identification approach described by Gasteiger et al. (2023) and applying the TECH-framework (Target user groups, Evaluation focus, Connectedness, Health domain)^56^. Detailed information on the search procedure is available in the published review protocol (no changes were made)^21^. The initial search was conducted on 29 July 2024 and was updated on 14th July 2025. To be included, applications were required to be permanently listed and approved for the treatment of either depression or GAD.

### Search strategy: studies

Eligible studies were identified through systematic searches of MEDLINE (via PubMed), Cochrane CENTRAL, and Web of Science. Publications in English or German were considered. To ensure comprehensiveness, the search was supplement by grey literature screening and backward citation tracking (snowballing).

A two-step screening process was conducted independently by two reviewers (SF and AS), involving initial screening of titles and abstracts, followed by full-text review. Discrepancies were resolved through discussion led by a third reviewer (AK). Inter-rater-reliability was assessed using Cohen’s kappa^57^. The initial database search was conducted on 30 July 2024 and updated on 14th July 2025. All references were imported into the Rayyan software to facilitate blinded screening and organization^58^.

The search strategy, including Medical Subject Headings (MeSH) and Boolean operators, was pre-specified in the registered review protocol (PROSPERO: CRD42024557629)^48^. Eligibility criteria were predefined according to the PICO (Population, Intervention, Comparison, Outcome) framework and applied consistently throughout the study selection process. The detailed eligibility criteria are provided in the study protocol^21^. Eligible studies included adults with depression (Population), evaluated DiGAs for depression approved for reimbursement under German statutory health insurance (Intervention), compared the intervention with a control condition (Comparison), and reported depressive symptom severity using validated outcome measures (Outcome)^21^. Studies were eligible irrespective of the country in which they were conducted, provided they evaluated an application that was permanently listed as a DiGA at the time of study selection. A PRISMA flow diagram was generated to illustrate the screening and inclusion process. The full search strategy for all databases including the number of hits is presented in Supplementary Material S3 Table S5.

### Data extraction

Data from eligible RCTs were independently extracted by two reviewers (AS, SF) using a piloted extraction form^21^. Discrepancies were resolved through discussion with a third reviewer (AK). In cases of missing or unclear information, study authors were contacted. Four authors responded and provided additional data.

Extracted variables included study characteristics (author, year, country, sample size), population demographics, intervention and comparator details, outcome measures and instruments, study design and methodology, duration, dropout rates, and main findings.

### Quality assessment

Two reviewers (SF and AS) independently used the Cochrane Risk of Bias 2 (RoB2) tool to assess the risk of bias of included RCTs^45^. The RoB2 compromises five categories: (1) bias arising from the randomization process; (2) bias due to deviations from intended interventions; (3) bias due to missing outcome data; (4) bias in measurement of the outcome and (5) bias in selection of the reported result. A third reviewer moderated any disagreements (AK). The overall quality of evidence was assessed using the GRADE (Grading of Recommendations, Assessment, Development and Evaluation) approach. Ratings were based on risk of bias, inconsistency, indirectness, imprecision, and publication bias^46^.

### Statistical analysis

All statistical analyses were conducted using R (version 4.1.0) and the ‘metafor’ package (version 4.8-0)^59,60^. Standardized mean differences (SMDs) between groups were calculated as the effect size metric, based on intention-to-treat (ITT) data, using both post-intervention and follow-up outcome measures. SMDs were computed as Hedges’ g, which corrects Cohen’s d for small sample bias. Negative values indicate improvement in symptoms. A random effects model was employed to account for heterogeneity across studies, which was anticipated due to variability in study design (e.g., intervention duration), intervention characteristics (different DiGA), and population features (e.g., comorbidities). Studies were weighted using the inverse-variance method and visually displayed in a forest plot. Heterogeneity was quantified using Higgins and Thompson’s I² statistic and tested using Cochran’s Q^61^. Potential publication bias was assessed visually via funnel plots and statistically using Egger’s regression test^62^. In addition, the Trim-and-Fill method and the Fail-Safe N procedure were applied to evaluate the potential impact of unpublished studies^63,64^.

To assess the robustness of the meta-analytic findings, several sensitivity analyses were performed. These included the use of alternative estimators for between-study variance (Sidik-Jonkmann, Paule-Mandl) as well as adjustments of variance (Hartung-Knapp-Sidik-Jonkman) and the exclusion of statistically influential outliers. Furthermore, a complementary sensitivity analysis was conducted based on per-protocol (PP) data.

Moderator analyses were performed to explore sources of heterogeneity. Pre-specified categorical moderators included intervention type, treatment setting (inpatient vs. outpatient), presence of comorbidities, and country. Evaluated continuous moderators included intervention duration (in weeks), mean participant age, proportion of female participants, and sample size.

## Supplementary Information

Below is the link to the electronic supplementary material.

Supplementary Material 1

Supplementary Material 2

Supplementary Material 3

## Data Availability

The protocol for the review is registered online in the International Prospective Register of Systematic Reviews (PROSEPRO [CRD42024557629](https://www.crd.york.ac.uk/PROSPERO/view/CRD42024557629)). The datasets and R-code are openly available at Open Science Framework: (https://osf.io/cf2q8/overview?view_only=99f739c3dab24ab58f0cbec97867bbda).
