# Supplementary material for: Efficacy of prescription-eligible digital health applications for depression and generalized anxiety disorder in Germany: a systematic review and meta-analysis
Source: Sci Rep. 2026 Sep 11;16:28490. doi: 10.1038/s41598-026-68875-y (PMC13569838; doi:10.1038/s41598-026-68875-y)
Supplement: Supplementary file 1 [file 41598_2026_68875_MOESM1_ESM.docx]

# **Supplementary information**

Supplementary figures

Digital Health Applications for Depression and Generalized Anxiety Disorder: A Systematic Review and Meta-analysis of Randomized Controlled Trials

A.Schmitz, S.Frey, A. Karimzadeh., A.L. Neves, A. Mayr, B. Weltermann

**Supplementary Figure 1: Supplementary Figure A. Identification of DiGA via BfArM directory.** PRISMA flow diagram outlining the identification process of digital health applications (DiGA) listed in the BfArM directory. The diagram shows the number of applications screened, excluded, and included based on predefined eligibility criteria. Only DiGA addressing depression or generalized anxiety disorder were included for synthesis.

**Supplementary Figure 2: Traffic light plot - Risk of bias assessment across included studies.** Risk of bias was evaluated using the Cochrane Risk of Bias 2 tool, covering five domains: D1 – randomisation process, D2 – deviations from intended interventions, D3 – missing outcome data, D4 – measurement of the outcome, and D5 – selection of the reported result. Each study was rated as having low risk, some concerns, or high risk of bias in each domain. Visual summaries reflect the overall methodological quality of the included randomized controlled trials.

**Supplementary Figure 3: Barplot - Risk of bias – percentage distribution.** Barplot showing the percentage of included randomized controlled trials rated as having low risk of bias, some concerns, or high risk of bias within each domain of the Cochrane Risk of Bias 2.0 tool. Colours indicate the level of risk (green = low, yellow = some concerns, red = high).

**Supplementary Figure 4: Forest-Plot - Effect of DiGAs on depressive symptoms at follow-up.**
Forest plot showing standardized mean differences between intervention and control groups in depressive symptom severity at follow-up. Estimates are based on randomized controlled trials evaluating DiGA for depression. Effect sizes were calculated using a fixed-effect model and based on intention-to-treat data where available. The plot displays individual study effects with 95% confidence intervals, as well as the pooled overall effect.

**Supplementary Figure 5: Funnel plot for follow-up effects of DiGAs on depressive symptoms.** Funnel plot assessing potential publication bias among randomized controlled trials evaluating digital health applications (DiGA) for depression at follow-up. Each point represents an individual study, plotted by its effect size and standard error. Symmetry of the plot was visually inspected to evaluate small-study effects.
