# Supplementary material for: Efficacy of prescription-eligible digital health applications for depression and generalized anxiety disorder in Germany: a systematic review and meta-analysis
Source: Sci Rep. 2026 Sep 11;16:28490. doi: 10.1038/s41598-026-68875-y (PMC13569838; doi:10.1038/s41598-026-68875-y)
Supplement: Supplementary file 2 [file 41598_2026_68875_MOESM3_ESM.docx]

# **Supplementary information**

Supplementary tables 2-5

Digital Health Applications for Depression and Generalized Anxiety Disorder: A Systematic Review and Meta-analysis of Randomized Controlled Trials

A.Schmitz, S.Frey, A. Karimzadeh., A.L. Neves, A. Mayr, B. Weltermann

**Supplementary Table S2. Results of categorical moderator analyses.** Summary of subgroup analyses examining the influence of categorical moderators on the effectiveness of DiGA interventions. For each moderator level, the table reports the number of contributing studies (with intervention and control group sizes), standardized mean difference (SMD), standard error (SE), Z-value, p-value, and 95% confidence intervals. The final columns present the test of moderators (QM, degrees of freedom [df], and p-value) and the test for residual heterogeneity (QE, df, and p-value), derived from random-effects models.

| **Moderator** | | **N of studies**  **(N_IG_/N_CG_)** | **SMD** | **SE** | **Zval** | **P** | **95 % CI** | **Test of moderators** | | | |
| --- | --- | --- | --- | --- | --- | --- | --- | --- | --- | --- | --- |
|  | | | | | | | | **QM(df)** | **p** | **QE(df)** | **P** |
| **Intervention (DiGA)** | | | | | | | |  |  |  |  |
|  | Deprexis | 12  (1,693/1,263) | -0.46 | 0.09 | -4.76 | < 0.0001 | [-0.65, -0.27] | 39.98 (3) | < 0.0001 | 65.55(14) | < 0.0001 |
|  | elona therapy / selfapys for depression | 2  (294/239) | -0.86 | 0.22 | -3.88 | 0.0001 | [-1.30, -0.43] |  |  |  |  |
|  | Novego | 3  (202/178) | -0.30 | 0.20 | -1.50 | 0.1349 | [-0.70, 0.09] |  |  |  |  |
| **Study design (Setting)** | | | | | | | |  |  |  |  |
|  | Inpatient | 2  (140/139) | -0.56 | 0.26 | -2.12 | 0.0338 | [-1.08,-0.04] | 0.09(1) | 0.76 | 80.94(14) | < 0.0001 |
|  | Outpatient | 15  (2,049/1,541) | -0.47 | 0.09 | -5.13 | < 0.0001 | [-0.66, -0.29] |  |  |  |  |
| **Sample characteristics (Comorbidity)** | | | | | | | |  |  |  |  |
|  | Dysthimia | 1  (25/26) | 0.65 | 0.43 | -1.51 | 0.1300 | [-1.50, 0.19] | 4.16(6) | 0.66 | 65.70(10) | < 0.0001 |
|  | Epilepsy | 1  (38/40) | -0.29 | 0.39 | -0.73 | 0.4626 | [-1.06, 0.48] |  |  |  |  |
|  | Gambling | 1  (71/69) | -0.06 | 0.36 | -0.18 | 0.8607 | [-0.78, 0.65] |  |  |  |  |
|  | Multiple sclerosis | 1  (45/45) | -0.32 | 0.39 | -0.84 | 0.4021 | [-1.08, 0.43] |  |  |  |  |
|  | None | 11  (1,908/1,402) | -0.57 | 0.11 | -5.38 | < 0.0001 | [-0.78, -0.37] |  |  |  |  |
|  | Pain | 1  (71/71) | -0.03 | 0.36 | -0.10 | 0.9220 | [-0.75, 0.68] |  |  |  |  |
|  | Schizophrenia | 1  (31/27) | -0.053 | 0.42 | -0.28 | 0.2015 | [1.36, 0.29] |  |  |  |  |
| **Study characteristics (Country)** | | | | | | | | | | | |
|  | Brazil | 1  (94/95) | -0.80 | 0.35 | -2.30 | 0.0212 | [-1.48, -0.12] | 2.30(3) | 0.51 | 65.95 (13) | < 0.0001 |
|  | Germany | 14  (1,785/1,468) | -0.43 | 0.10 | -4.51 | < 0.0001 | [-0.61, -0.24) |  |  |  |  |
|  | Switzerland | 1  (25/26) | -0.65 | 0.42 | -1.54 | 0.1229 | [-1.48, 0.18] |  |  |  |  |
|  | United States | 1  (285/91) | -0.81 | 0.34 | .2.43 | 0.0151 | [-1.47, -0.16] |  |  |  |  |

**Supplementary Table S3. Results of continuous moderator analyses. Summary of random-effects meta-regression analyses examining the relationship between continuous study-level variables and the effectiveness of DiGA interventions. For each moderator, the table reports the standardized mean difference (SMD, standard error (SE), Z-value, p-value, and 95% confidence interval (CI). The test of moderators indicates the significance of the overall meta-regression model. Measures of heterogeneity (I²) and proportion of explained variance (R²) are provided.**

| **Variable** | **SMD** | **SE** | **Zval** | **P** | **95% CI** | **Test of Moderators** | **I^2^** | **R^2^** |
| --- | --- | --- | --- | --- | --- | --- | --- | --- |
| DiGA-duration (in weeks) | -0.05 | 0.05 | -1.08 | 0.28 | [-0.15, 0.04] | QM(df = 1) = 0.64, p = 0.42 | 81.23% | 0.00% |
| Average Age (in years) | 0.01 | 0.02 | 0.36 | 0.72 | [-0.03, 0.04] | QM(df = 1) = 0.13, p = 0.72 | 81.54% | 0.00% |
| Proportion of female participants | -0.01 | 0.01 | -1.12 | 0.26 | [-0.02, 0.00] | QM(df = 1) = 1.26, p = 0.26 | 81.30% | 4.79% |
| Sample size | -0.00 | 0.00 | 0.03 | 0.97 | [-0.00, 0.00] | QM(df = 1) = 0.00, p = 0.97 | 80.05% | 0.00% |

**Supplementary Table S4.** **RISMA 2020 checklist for reporting of the systematic review and meta-analysis.** Completed Preferred Reporting Items for Systematic Reviews and Meta-Analyses (PRISMA) 2020 checklist indicating where each reporting item is addressed in the manuscript. Page numbers refer to locations in the main text or supplementary materials where each item is covered.

| **Section and Topic** | **Item #** | **Checklist item** | **Location where item is reported** |
| --- | --- | --- | --- |
| **TITLE** | | |  |
| Title | 1 | Identify the report as a systematic review. | p. 1 l. 1f |
| **ABSTRACT** | | |  |
| Abstract | 2 | See the PRISMA 2020 for Abstracts checklist. | p. 2 |
| **INTRODUCTION** | | |  |
| Rationale | 3 | Describe the rationale for the review in the context of existing knowledge. | p.4 l 80ff |
| Objectives | 4 | Provide an explicit statement of the objective(s) or question(s) the review addresses. | p. 4 l. 85ff |
| **METHODS** | | |  |
| Eligibility criteria | 5 | Specify the inclusion and exclusion criteria for the review and how studies were grouped for the syntheses. | p. 26 l. 360ff |
| Information sources | 6 | Specify all databases, registers, websites, organisations, reference lists and other sources searched or consulted to identify studies. Specify the date when each source was last searched or consulted. | p. 26 l. 360ff |
| Search strategy | 7 | Present the full search strategies for all databases, registers and websites, including any filters and limits used. | Supplementary Table S5 |
| Selection process | 8 | Specify the methods used to decide whether a study met the inclusion criteria of the review, including how many reviewers screened each record and each report retrieved, whether they worked independently, and if applicable, details of automation tools used in the process. | p. 26 l. 360ff |
| Data collection process | 9 | Specify the methods used to collect data from reports, including how many reviewers collected data from each report, whether they worked independently, any processes for obtaining or confirming data from study investigators, and if applicable, details of automation tools used in the process. | p. 27 l. 376ff |
| Data items | 10a | List and define all outcomes for which data were sought. Specify whether all results that were compatible with each outcome domain in each study were sought (e.g. for all measures, time points, analyses), and if not, the methods used to decide which results to collect. | p. 27 l. 380ff |
|  | 10b | List and define all other variables for which data were sought (e.g. participant and intervention characteristics, funding sources). Describe any assumptions made about any missing or unclear information. | p. 27 l. 380ff |
| Study risk of bias assessment | 11 | Specify the methods used to assess risk of bias in the included studies, including details of the tool(s) used, how many reviewers assessed each study and whether they worked independently, and if applicable, details of automation tools used in the process. | p. 27 l. 384ff |
| Effect measures | 12 | Specify for each outcome the effect measure(s) (e.g. risk ratio, mean difference) used in the synthesis or presentation of results. | p. 27 l. 392ff |
| Synthesis methods | 13a | Describe the processes used to decide which studies were eligible for each synthesis (e.g. tabulating the study intervention characteristics and comparing against the planned groups for each synthesis (item #5)). | p. 27 l. 392ff |
|  | 13b | Describe any methods required to prepare the data for presentation or synthesis, such as handling of missing summary statistics, or data conversions. | p. 27 l. 392ff |
|  | 13c | Describe any methods used to tabulate or visually display results of individual studies and syntheses. | p. 27 l. 392ff |
|  | 13d | Describe any methods used to synthesize results and provide a rationale for the choice(s). If meta-analysis was performed, describe the model(s), method(s) to identify the presence and extent of statistical heterogeneity, and software package(s) used. | p. 27 l. 392ff |
|  | 13e | Describe any methods used to explore possible causes of heterogeneity among study results (e.g. subgroup analysis, meta-regression). | p. 27 l. 392ff |
|  | 13f | Describe any sensitivity analyses conducted to assess robustness of the synthesized results. | p. 27 l. 392ff |
| Reporting bias assessment | 14 | Describe any methods used to assess risk of bias due to missing results in a synthesis (arising from reporting biases). | p. 27 l. 392ff |
| Certainty assessment | 15 | Describe any methods used to assess certainty (or confidence) in the body of evidence for an outcome. | p. 27 l. 380ff |
| **RESULTS** | | |  |
| Study selection | 16a | Describe the results of the search and selection process, from the number of records identified in the search to the number of studies included in the review, ideally using a flow diagram. | p. 6 l. 114 |
|  | 16b | Cite studies that might appear to meet the inclusion criteria, but which were excluded, and explain why they were excluded. | p- 5 l. 107f |
| Study characteristics | 17 | Cite each included study and present its characteristics. | Table 1 |
| Risk of bias in studies | 18 | Present assessments of risk of bias for each included study. | Supplementary Fig. S2 / S3 |
| Results of individual studies | 19 | For all outcomes, present, for each study: (a) summary statistics for each group (where appropriate) and (b) an effect estimate and its precision (e.g. confidence/credible interval), ideally using structured tables or plots. | Figure 2 |
| Results of syntheses | 20a | For each synthesis, briefly summarise the characteristics and risk of bias among contributing studies. | p. 16 l. 153 |
|  | 20b | Present results of all statistical syntheses conducted. If meta-analysis was done, present for each the summary estimate and its precision (e.g. confidence/credible interval) and measures of statistical heterogeneity. If comparing groups, describe the direction of the effect. | p. 16 l. 171 |
|  | 20c | Present results of all investigations of possible causes of heterogeneity among study results. | p. 20 l. 227ff |
|  | 20d | Present results of all sensitivity analyses conducted to assess the robustness of the synthesized results. | p. 18 l. 204ff |
| Reporting biases | 21 | Present assessments of risk of bias due to missing results (arising from reporting biases) for each synthesis assessed. | p. 17 l. 190ff |
| Certainty of evidence | 22 | Present assessments of certainty (or confidence) in the body of evidence for each outcome assessed. | p. 22 l. 275ff |
| **DISCUSSION** | | |  |
| Discussion | 23a | Provide a general interpretation of the results in the context of other evidence. | p. 23 l. 288ff |
|  | 23b | Discuss any limitations of the evidence included in the review. | p. 24 l. 297ff |
|  | 23c | Discuss any limitations of the review processes used. | p. 25 l. 325ff |
|  | 23d | Discuss implications of the results for practice, policy, and future research. | p. 25 l. 334ff |
| **OTHER INFORMATION** | | |  |
| Registration and protocol | 24a | Provide registration information for the review, including register name and registration number, or state that the review was not registered. | p. 28 l. 413ff |
|  | 24b | Indicate where the review protocol can be accessed, or state that a protocol was not prepared. | p. 28 l. 413ff |
|  | 24c | Describe and explain any amendments to information provided at registration or in the protocol. | p. 28 l. 413ff |
| Support | 25 | Describe sources of financial or non-financial support for the review, and the role of the funders or sponsors in the review. | p. 37 |
| Competing interests | 26 | Declare any competing interests of review authors. | p. 37 |
| Availability of data, code and other materials | 27 | Report which of the following are publicly available and where they can be found: template data collection forms; data extracted from included studies; data used for all analyses; analytic code; any other materials used in the review. | p. 28 l. 413ff |

Page MJ, McKenzie JE, Bossuyt PM, Boutron I, Hoffmann TC, Mulrow CD, Shamseer L, Tetzlaff JM, Akl EA, Brennan SE, Chou R. The PRISMA 2020 statement: an updated guideline for reporting systematic reviews. Bmj. 2021 Mar 29;372:n71. doi: <https://doi.org/10.1136/bmj.n71>

**Supplementary Table S5. Full search strategies used across databases and for DiGA identification.**
Complete list of search terms and Boolean operators used for all electronic databases included in the systematic review. Search strategies were tailored to each database’s syntax and include controlled vocabulary (e.g., MeSH terms). The date of the last search and all search limits applied are reported.

| **CENTRAL – Date Run: 30/07/2024 16:40:12** | | |
| --- | --- | --- |
| ID | Search | Hits |
| #1 | (Deprexis):ti,ab,kw (Word variations have been searched) | 61 |
| #2 | effect OR effecting OR effective OR effectively OR effectiveness OR effectivenesses OR effectives OR effectivities OR effectivity OR effects OR effectiv | 1288807 |
| #3 | patient safety OR patient AND safety OR patient safety OR patient harm OR patient AND harm OR patient harm OR safety management | 123223 |
| #4 | equities OR equity OR disparit OR inequit OR inequalit | 2251 |
| #5 | studies OR study OR study s OR studying OR studys OR research personnel OR research AND personnel OR research personnel OR researcher OR researchers OR research OR research s OR researchable OR researche OR researched OR researchers OR researches OR researching OR researchs OR clinical trial OR clinical trials as topic OR clinical trial OR randomized controlled trial OR randomized controlled trials as topic OR randomized controlled trial OR randomised controlled trial | 1865550 |
| #6 | #1 AND #5 AND (#2 OR #3 OR #4) | 46 |
| **CENTRAL - Date Run: 14/07/2025 12:08:04** | | |
| ID | Search | Hits |
| #1 | Deprexis | 63 |
| #2 | effect OR effecting OR effective OR effectively OR effectiveness OR effectivenesses OR effectives OR effectivities OR effectivity OR effects OR effectiv | 1342628 |
| #3 | patient safety OR patient AND safety OR patient safety OR patient harm OR patient AND harm OR patient harm OR safety management | 128734 |
| #4 | equities OR equity OR disparit OR inequit OR inequalit | 2815 |
| #5 | studies OR study OR study s OR studying OR studys OR research personnel OR research AND personnel OR research personnel OR researcher OR researchers OR research OR research s OR researchable OR researche OR researched OR researchers OR researches OR researching OR researchs OR clinical trial OR clinical trials as topic OR clinical trial OR randomized controlled trial OR randomized controlled trials as topic OR randomized controlled trial OR randomised controlled trial | 1951451 |
| #6 | #1 AND #5 AND (#2 OR #3 OR #4) | 48 |
| **Pubmed - Date Run: 30/07/2024 15:28:09** | | |
| 6 | #1 AND #5 AND (#2 OR #3 OR #4),Most Recent,,"""Deprexis""[All Fields] AND (""studies""[All Fields] OR ""study""[All Fields] OR ""study s""[All Fields] OR ""studying""[All Fields] OR ""studys""[All Fields] OR (""research personnel""[MeSH Terms] OR (""research""[All Fields] AND ""personnel""[All Fields]) OR ""research personnel""[All Fields] OR ""researcher""[All Fields] OR ""researchers""[All Fields] OR ""research""[MeSH Terms] OR ""research""[All Fields] OR ""research s""[All Fields] OR ""researchable""[All Fields] OR ""researche""[All Fields] OR ""researched""[All Fields] OR ""researcher s""[All Fields] OR ""researches""[All Fields] OR ""researching""[All Fields] OR ""researchs""[All Fields]) OR (""clinical trial""[Publication Type] OR ""clinical trials as topic""[MeSH Terms] OR ""clinical trial""[All Fields]) OR (""randomized controlled trial""[Publication Type] OR ""randomized controlled trials as topic""[MeSH Terms] OR ""randomized controlled trial""[All Fields] OR ""randomised controlled trial""[All Fields])) AND (""effect""[All Fields] OR ""effecting""[All Fields] OR ""effective""[All Fields] OR ""effectively""[All Fields] OR ""effectiveness""[All Fields] OR ""effectivenesses""[All Fields] OR ""effectives""[All Fields] OR ""effectivities""[All Fields] OR ""effectivity""[All Fields] OR ""effects""[All Fields] OR ""effectiv*""[All Fields] OR (""patient safety""[MeSH Terms] OR (""patient""[All Fields] AND ""safety""[All Fields]) OR ""patient safety""[All Fields] OR (""patient harm""[MeSH Terms] OR (""patient""[All Fields] AND ""harm""[All Fields]) OR ""patient harm""[All Fields]) OR ""safety management""[MeSH Terms]) OR (""equities""[All Fields] OR ""equity""[All Fields] OR ""disparit*""[All Fields] OR ""inequit*""[All Fields] OR ""inequalit*""[All Fields]))" | 45 |
| 5 | "(""studies""[All Fields] OR ""study""[All Fields] OR ""study s""[All Fields] OR ""studying""[All Fields] OR ""studys""[All Fields] OR (""research personnel""[MeSH Terms] OR (""research""[All Fields] AND ""personnel""[All Fields]) OR ""research personnel""[All Fields] OR ""researcher""[All Fields] OR ""researchers""[All Fields] OR ""research""[MeSH Terms] OR ""research""[All Fields] OR ""research s""[All Fields] OR ""researchable""[All Fields] OR ""researche""[All Fields] OR ""researched""[All Fields] OR ""researcher s""[All Fields] OR ""researches""[All Fields] OR ""researching""[All Fields] OR ""researchs""[All Fields]) OR (""clinical trial""[Publication Type] OR ""clinical trials as topic""[MeSH Terms] OR ""clinical trial""[All Fields]) OR (""randomized controlled trial""[Publication Type] OR ""randomized controlled trials as topic""[MeSH Terms] OR ""randomized controlled trial""[All Fields] OR ""randomised controlled trial""[All Fields]))",Most Recent,,"""studies""[All Fields] OR ""study""[All Fields] OR ""study s""[All Fields] OR ""studying""[All Fields] OR ""studys""[All Fields] OR (""research personnel""[MeSH Terms] OR (""research""[All Fields] AND ""personnel""[All Fields]) OR ""research personnel""[All Fields] OR ""researcher""[All Fields] OR ""researchers""[All Fields] OR ""research""[MeSH Terms] OR ""research""[All Fields] OR ""research s""[All Fields] OR ""researchable""[All Fields] OR ""researche""[All Fields] OR ""researched""[All Fields] OR ""researcher s""[All Fields] OR ""researches""[All Fields] OR ""researching""[All Fields] OR ""researchs""[All Fields]) OR (""clinical trial""[Publication Type] OR ""clinical trials as topic""[MeSH Terms] OR ""clinical trial""[All Fields]) OR (""randomized controlled trial""[Publication Type] OR ""randomized controlled trials as topic""[MeSH Terms] OR ""randomized controlled trial""[All Fields] OR ""randomised controlled trial""[All Fields])" | 22,574,360 |
| 4 | "(""equities""[All Fields] OR ""equity""[All Fields] OR ""disparit*""[All Fields] OR ""inequit*""[All Fields] OR ""inequalit*""[All Fields])",Most Recent,,"""equities""[All Fields] OR ""equity""[All Fields] OR ""disparit*""[All Fields] OR ""inequit*""[All Fields] OR ""inequalit*""[All Fields]" | 273,910 |
| 3 | 3,"(""patient safety""[MeSH Terms] OR (""patient""[All Fields] AND ""safety""[All Fields]) OR ""patient safety""[All Fields] OR (""patient harm""[MeSH Terms] OR (""patient""[All Fields] AND ""harm""[All Fields]) OR ""patient harm""[All Fields]) OR ""safety management""[MeSH Terms])",Most Recent,,"""patient safety""[MeSH Terms] OR (""patient""[All Fields] AND ""safety""[All Fields]) OR ""patient safety""[All Fields] OR (""patient harm""[MeSH Terms] OR (""patient""[All Fields] AND ""harm""[All Fields]) OR ""patient harm""[All Fields]) OR ""safety management""[MeSH Terms]" | 253,755 |
| 2 | "(""effect""[All Fields] OR ""effecting""[All Fields] OR ""effective""[All Fields] OR ""effectively""[All Fields] OR ""effectiveness""[All Fields] OR ""effectivenesses""[All Fields] OR ""effectives""[All Fields] OR ""effectivities""[All Fields] OR ""effectivity""[All Fields] OR ""effects""[All Fields] OR ""effectiv*""[All Fields])",Most Recent,,"""effect""[All Fields] OR ""effecting""[All Fields] OR ""effective""[All Fields] OR ""effectively""[All Fields] OR ""effectiveness""[All Fields] OR ""effectivenesses""[All Fields] OR ""effectives""[All Fields] OR ""effectivities""[All Fields] OR ""effectivity""[All Fields] OR ""effects""[All Fields] OR ""effectiv*""[All Fields]" | 11,682,789 |
| 1 | Deprexis,Most Recent,,"""Deprexis""[All Fields]" | 61 |
| **Pubmed - Date Run: 14/07/2025 15:55:49** | | |
| ID | Search | Hits |
| 6 | #1 AND #5 AND (#2 OR #3 OR #4),Most Recent,,"""Deprexis""[All Fields] AND (""studies""[All Fields] OR ""study""[All Fields] OR ""study s""[All Fields] OR ""studying""[All Fields] OR ""studys""[All Fields] OR (""research personnel""[MeSH Terms] OR (""research""[All Fields] AND ""personnel""[All Fields]) OR ""research personnel""[All Fields] OR ""researcher""[All Fields] OR ""researchers""[All Fields] OR ""research""[MeSH Terms] OR ""research""[All Fields] OR ""research s""[All Fields] OR ""researchable""[All Fields] OR ""researche""[All Fields] OR ""researched""[All Fields] OR ""researcher s""[All Fields] OR ""researches""[All Fields] OR ""researching""[All Fields] OR ""researchs""[All Fields]) OR (""clinical trial""[Publication Type] OR ""clinical trials as topic""[MeSH Terms] OR ""clinical trial""[All Fields]) OR (""randomized controlled trial""[Publication Type] OR ""randomized controlled trials as topic""[MeSH Terms] OR ""randomized controlled trial""[All Fields] OR ""randomised controlled trial""[All Fields])) AND (""effect""[All Fields] OR ""effecting""[All Fields] OR ""effective""[All Fields] OR ""effectively""[All Fields] OR ""effectiveness""[All Fields] OR ""effectivenesses""[All Fields] OR ""effectives""[All Fields] OR ""effectivities""[All Fields] OR ""effectivity""[All Fields] OR ""effects""[All Fields] OR ""effectiv*""[All Fields] OR (""patient safety""[MeSH Terms] OR (""patient""[All Fields] AND ""safety""[All Fields]) OR ""patient safety""[All Fields] OR (""patient harm""[MeSH Terms] OR (""patient""[All Fields] AND ""harm""[All Fields]) OR ""patient harm""[All Fields]) OR ""safety management""[MeSH Terms]) OR (""equities""[All Fields] OR ""equity""[All Fields] OR ""disparit*""[All Fields] OR ""inequit*""[All Fields] OR ""inequalit*""[All Fields]))" | 47 |
| 5 | "(""studies""[All Fields] OR ""study""[All Fields] OR ""study s""[All Fields] OR ""studying""[All Fields] OR ""studys""[All Fields] OR (""research personnel""[MeSH Terms] OR (""research""[All Fields] AND ""personnel""[All Fields]) OR ""research personnel""[All Fields] OR ""researcher""[All Fields] OR ""researchers""[All Fields] OR ""research""[MeSH Terms] OR ""research""[All Fields] OR ""research s""[All Fields] OR ""researchable""[All Fields] OR ""researche""[All Fields] OR ""researched""[All Fields] OR ""researcher s""[All Fields] OR ""researches""[All Fields] OR ""researching""[All Fields] OR ""researchs""[All Fields]) OR (""clinical trial""[Publication Type] OR ""clinical trials as topic""[MeSH Terms] OR ""clinical trial""[All Fields]) OR (""randomized controlled trial""[Publication Type] OR ""randomized controlled trials as topic""[MeSH Terms] OR ""randomized controlled trial""[All Fields] OR ""randomised controlled trial""[All Fields])",Most Recent,,"""studies""[All Fields] OR ""study""[All Fields] OR ""study s""[All Fields] OR ""studying""[All Fields] OR ""studys""[All Fields] OR (""research personnel""[MeSH Terms] OR (""research""[All Fields] AND ""personnel""[All Fields]) OR ""research personnel""[All Fields] OR ""researcher""[All Fields] OR ""researchers""[All Fields] OR ""research""[MeSH Terms] OR ""research""[All Fields] OR ""research s""[All Fields] OR ""researchable""[All Fields] OR ""researche""[All Fields] OR ""researched""[All Fields] OR ""researcher s""[All Fields] OR ""researches""[All Fields] OR ""researching""[All Fields] OR ""researchs""[All Fields]) OR (""clinical trial""[Publication Type] OR ""clinical trials as topic""[MeSH Terms] OR ""clinical trial""[All Fields]) OR (""randomized controlled trial""[Publication Type] OR ""randomized controlled trials as topic""[MeSH Terms] OR ""randomized controlled trial""[All Fields] OR ""randomised controlled trial""[All Fields])" | 23,805,719 |
| 4 | "(""equities""[All Fields] OR ""equity""[All Fields] OR ""disparit*""[All Fields] OR ""inequit*""[All Fields] OR ""inequalit*""[All Fields])",Most Recent,,"""equities""[All Fields] OR ""equity""[All Fields] OR ""disparit*""[All Fields] OR ""inequit*""[All Fields] OR ""inequalit*""[All Fields]" | 317,276 |
| 3 | (""patient safety""[MeSH Terms] OR (""patient""[All Fields] AND ""safety""[All Fields]) OR ""patient safety""[All Fields] OR (""patient harm""[MeSH Terms] OR (""patient""[All Fields] AND ""harm""[All Fields]) OR ""patient harm""[All Fields]) OR ""safety management""[MeSH Terms])",Most Recent,,"""patient safety""[MeSH Terms] OR (""patient""[All Fields] AND ""safety""[All Fields]) OR ""patient safety""[All Fields] OR (""patient harm""[MeSH Terms] OR (""patient""[All Fields] AND ""harm""[All Fields]) OR ""patient harm""[All Fields]) OR ""safety management""[MeSH Terms]" | 278,587", |
| 2 | (""effect""[All Fields] OR ""effecting""[All Fields] OR ""effective""[All Fields] OR ""effectively""[All Fields] OR ""effectiveness""[All Fields] OR ""effectivenesses""[All Fields] OR ""effectives""[All Fields] OR ""effectivities""[All Fields] OR ""effectivity""[All Fields] OR ""effects""[All Fields] OR ""effectiv*""[All Fields])",Most Recent,,"""effect""[All Fields] OR ""effecting""[All Fields] OR ""effective""[All Fields] OR ""effectively""[All Fields] OR ""effectiveness""[All Fields] OR ""effectivenesses""[All Fields] OR ""effectives""[All Fields] OR ""effectivities""[All Fields] OR ""effectivity""[All Fields] OR ""effects""[All Fields] OR ""effectiv*""[All Fields]" | 12,315,133 |
| 1 | Deprexis,Most Recent,,"""Deprexis""[All Fields]" | 65 |
| **Web of Science - Date Run: 30/07/2024 13:53:50** | | |
| ID | Search | Hits |
| 1 | ALL=(Deprexis) | 65 |
| 2 | ALL=((effect OR effective OR effectively OR effectiveness OR effects OR effectiv*)) | 15644528 |
| 3 | ALL=((patient safety OR patient harm OR safety management)) | 682985 |
| 4 | ALL=((equities OR equity OR disparit* OR inequit* OR inequalit*)) | 636039 |
| 5 | ALL=((studies OR study OR studying OR studys OR research personnel OR research OR researcher OR researchers OR clinical trial OR clinical trials as topic OR clinical trial OR randomized controlled trial OR randomized controlled trials as topic OR randomized controlled trial OR randomised controlled trial)) | 38704259 |
| 6 | #1 AND #5 AND (#2 OR #3 OR #4) | 53 |
| **Web of Science - Date Run: 14/07/2025 13:23:23** | | |
| ID | Search | Hits |
| 1 | ALL=(Deprexis) | 71 |
| 2 | ALL=(((effect OR effective OR effectively OR effectiveness OR effects OR effectiv*))) | 16777749 |
| 3 | ALL=((patient safety OR patient harm OR safety management)) | 758088 |
| 4 | ALL=((equities OR equity OR disparit* OR inequit* OR inequalit*)) | 713759 |
| 5 | ALL=((studies OR study OR studying OR studys OR research personnel OR research OR researcher OR researchers OR clinical trial OR clinical trials as topic OR clinical trial OR randomized controlled trial OR randomized controlled trials as topic OR randomized controlled trial OR randomised controlled trial)) | 41244021 |
| 6 | #1 AND #5 AND (#2 OR #3 OR #4) | 57 |

| **CENTRAL - Date Run: 30/07/2024 22:11:54** | | |
| --- | --- | --- |
| ID | Search | Hits |
| #1 | edupression.com | 2 |
| #2 | effect OR effecting OR effective OR effectively OR effectiveness OR effectivenesses OR effectives OR effectivities OR effectivity OR effects OR effectiv | 1288807 |
| #3 | patient safety OR patient AND safety OR patient safety OR patient harm OR patient AND harm OR patient harm OR safety management | 123223 |
| #4 | equities OR equity OR disparit OR inequit OR inequalit | 2251 |
| #5 | studies OR study OR study s OR studying OR studys OR research personnel OR research AND personnel OR research personnel OR researcher OR researchers OR research OR research s OR researchable OR researche OR researched OR researchers OR researches OR researching OR researchs OR clinical trial OR clinical trials as topic OR clinical trial OR randomized controlled trial OR randomized controlled trials as topic OR randomized controlled trial OR randomised controlled trial | 1865550 |
| #6 | #1 #5 AND (#2 OR #3 OR #4) | 1 |
| **CENTRAL - Date Run: 14/07/2025 12:06:49** | | |
| ID | Search | Hits |
| #1 | edupression.com | 2 |
| #2 | effect OR effecting OR effective OR effectively OR effectiveness OR effectivenesses OR effectives OR effectivities OR effectivity OR effects OR effectiv | 1342628 |
| #3 | patient safety OR patient AND safety OR patient safety OR patient harm OR patient AND harm OR patient harm OR safety management | 128734 |
| #4 | equities OR equity OR disparit OR inequit OR inequalit | 2815 |
| #5 | studies OR study OR study s OR studying OR studys OR research personnel OR research AND personnel OR research personnel OR researcher OR researchers OR research OR research s OR researchable OR researche OR researched OR researchers OR researches OR researching OR researchs OR clinical trial OR clinical trials as topic OR clinical trial OR randomized controlled trial OR randomized controlled trials as topic OR randomized controlled trial OR randomised controlled trial | 1951451 |
| #6 | #1 AND #5 AND (#2 OR #3 OR #4) | 1 |
| **Pubmed - Date Run: 30/07/2024 09:40:28** | | |
| ID | Search | Hits |
| 6 | #1 AND #5 AND (#2 OR #3 OR #4),Most Recent,,"""edupression.com""[All Fields] AND (""studies""[All Fields] OR ""study""[All Fields] OR ""study s""[All Fields] OR ""studying""[All Fields] OR ""studys""[All Fields] OR (""research personnel""[MeSH Terms] OR (""research""[All Fields] AND ""personnel""[All Fields]) OR ""research personnel""[All Fields] OR ""researcher""[All Fields] OR ""researchers""[All Fields] OR ""research""[MeSH Terms] OR ""research""[All Fields] OR ""research s""[All Fields] OR ""researchable""[All Fields] OR ""researche""[All Fields] OR ""researched""[All Fields] OR ""researcher s""[All Fields] OR ""researches""[All Fields] OR ""researching""[All Fields] OR ""researchs""[All Fields]) OR (""clinical trial""[Publication Type] OR ""clinical trials as topic""[MeSH Terms] OR ""clinical trial""[All Fields]) OR (""randomized controlled trial""[Publication Type] OR ""randomized controlled trials as topic""[MeSH Terms] OR ""randomized controlled trial""[All Fields] OR ""randomised controlled trial""[All Fields])) AND (""effect""[All Fields] OR ""effecting""[All Fields] OR ""effective""[All Fields] OR ""effectively""[All Fields] OR ""effectiveness""[All Fields] OR ""effectivenesses""[All Fields] OR ""effectives""[All Fields] OR ""effectivities""[All Fields] OR ""effectivity""[All Fields] OR ""effects""[All Fields] OR ""effectiv*""[All Fields] OR (""patient safety""[MeSH Terms] OR (""patient""[All Fields] AND ""safety""[All Fields]) OR ""patient safety""[All Fields] OR (""patient harm""[MeSH Terms] OR (""patient""[All Fields] AND ""harm""[All Fields]) OR ""patient harm""[All Fields]) OR ""safety management""[MeSH Terms]) OR (""equities""[All Fields] OR ""equity""[All Fields] OR ""disparit*""[All Fields] OR ""inequit*""[All Fields] OR ""inequalit*""[All Fields]))" | 0 |
| 5 | "(""studies""[All Fields] OR ""study""[All Fields] OR ""study s""[All Fields] OR ""studying""[All Fields] OR ""studys""[All Fields] OR (""research personnel""[MeSH Terms] OR (""research""[All Fields] AND ""personnel""[All Fields]) OR ""research personnel""[All Fields] OR ""researcher""[All Fields] OR ""researchers""[All Fields] OR ""research""[MeSH Terms] OR ""research""[All Fields] OR ""research s""[All Fields] OR ""researchable""[All Fields] OR ""researche""[All Fields] OR ""researched""[All Fields] OR ""researcher s""[All Fields] OR ""researches""[All Fields] OR ""researching""[All Fields] OR ""researchs""[All Fields]) OR (""clinical trial""[Publication Type] OR ""clinical trials as topic""[MeSH Terms] OR ""clinical trial""[All Fields]) OR (""randomized controlled trial""[Publication Type] OR ""randomized controlled trials as topic""[MeSH Terms] OR ""randomized controlled trial""[All Fields] OR ""randomised controlled trial""[All Fields]))",Most Recent,,"""studies""[All Fields] OR ""study""[All Fields] OR ""study s""[All Fields] OR ""studying""[All Fields] OR ""studys""[All Fields] OR (""research personnel""[MeSH Terms] OR (""research""[All Fields] AND ""personnel""[All Fields]) OR ""research personnel""[All Fields] OR ""researcher""[All Fields] OR ""researchers""[All Fields] OR ""research""[MeSH Terms] OR ""research""[All Fields] OR ""research s""[All Fields] OR ""researchable""[All Fields] OR ""researche""[All Fields] OR ""researched""[All Fields] OR ""researcher s""[All Fields] OR ""researches""[All Fields] OR ""researching""[All Fields] OR ""researchs""[All Fields]) OR (""clinical trial""[Publication Type] OR ""clinical trials as topic""[MeSH Terms] OR ""clinical trial""[All Fields]) OR (""randomized controlled trial""[Publication Type] OR ""randomized controlled trials as topic""[MeSH Terms] OR ""randomized controlled trial""[All Fields] OR ""randomised controlled trial""[All Fields])" | 22,574,360 |
| 4 | "(""equities""[All Fields] OR ""equity""[All Fields] OR ""disparit*""[All Fields] OR ""inequit*""[All Fields] OR ""inequalit*""[All Fields])",Most Recent,,"""equities""[All Fields] OR ""equity""[All Fields] OR ""disparit*""[All Fields] OR ""inequit*""[All Fields] OR ""inequalit*""[All Fields]" | 317,276 |
| 3 | 3,"(""patient safety""[MeSH Terms] OR (""patient""[All Fields] AND ""safety""[All Fields]) OR ""patient safety""[All Fields] OR (""patient harm""[MeSH Terms] OR (""patient""[All Fields] AND ""harm""[All Fields]) OR ""patient harm""[All Fields]) OR ""safety management""[MeSH Terms])",Most Recent,,"""patient safety""[MeSH Terms] OR (""patient""[All Fields] AND ""safety""[All Fields]) OR ""patient safety""[All Fields] OR (""patient harm""[MeSH Terms] OR (""patient""[All Fields] AND ""harm""[All Fields]) OR ""patient harm""[All Fields]) OR ""safety management""[MeSH Terms]" | 278,587", |
| 2 | "(""effect""[All Fields] OR ""effecting""[All Fields] OR ""effective""[All Fields] OR ""effectively""[All Fields] OR ""effectiveness""[All Fields] OR ""effectivenesses""[All Fields] OR ""effectives""[All Fields] OR ""effectivities""[All Fields] OR ""effectivity""[All Fields] OR ""effects""[All Fields] OR ""effectiv*""[All Fields])",Most Recent,,"""effect""[All Fields] OR ""effecting""[All Fields] OR ""effective""[All Fields] OR ""effectively""[All Fields] OR ""effectiveness""[All Fields] OR ""effectivenesses""[All Fields] OR ""effectives""[All Fields] OR ""effectivities""[All Fields] OR ""effectivity""[All Fields] OR ""effects""[All Fields] OR ""effectiv*""[All Fields]" | 12,315,133 |
| 1 | edupression.com - Schema: all,Most Recent,,edupression.com | 0 |
| **Pubmed - Date Run: 14/07/2025 16:48:02** | | |
| ID | Search | Hits |
| 6 | #1 AND #5 AND (#2 OR #3 OR #4),Most Recent,,"""edupression.com""[All Fields] AND (""studies""[All Fields] OR ""study""[All Fields] OR ""study s""[All Fields] OR ""studying""[All Fields] OR ""studys""[All Fields] OR (""research personnel""[MeSH Terms] OR (""research""[All Fields] AND ""personnel""[All Fields]) OR ""research personnel""[All Fields] OR ""researcher""[All Fields] OR ""researchers""[All Fields] OR ""research""[MeSH Terms] OR ""research""[All Fields] OR ""research s""[All Fields] OR ""researchable""[All Fields] OR ""researche""[All Fields] OR ""researched""[All Fields] OR ""researcher s""[All Fields] OR ""researches""[All Fields] OR ""researching""[All Fields] OR ""researchs""[All Fields]) OR (""clinical trial""[Publication Type] OR ""clinical trials as topic""[MeSH Terms] OR ""clinical trial""[All Fields]) OR (""randomized controlled trial""[Publication Type] OR ""randomized controlled trials as topic""[MeSH Terms] OR ""randomized controlled trial""[All Fields] OR ""randomised controlled trial""[All Fields])) AND (""effect""[All Fields] OR ""effecting""[All Fields] OR ""effective""[All Fields] OR ""effectively""[All Fields] OR ""effectiveness""[All Fields] OR ""effectivenesses""[All Fields] OR ""effectives""[All Fields] OR ""effectivities""[All Fields] OR ""effectivity""[All Fields] OR ""effects""[All Fields] OR ""effectiv*""[All Fields] OR (""patient safety""[MeSH Terms] OR (""patient""[All Fields] AND ""safety""[All Fields]) OR ""patient safety""[All Fields] OR (""patient harm""[MeSH Terms] OR (""patient""[All Fields] AND ""harm""[All Fields]) OR ""patient harm""[All Fields]) OR ""safety management""[MeSH Terms]) OR (""equities""[All Fields] OR ""equity""[All Fields] OR ""disparit*""[All Fields] OR ""inequit*""[All Fields] OR ""inequalit*""[All Fields]))" | 0 |
| 5 | "(""studies""[All Fields] OR ""study""[All Fields] OR ""study s""[All Fields] OR ""studying""[All Fields] OR ""studys""[All Fields] OR (""research personnel""[MeSH Terms] OR (""research""[All Fields] AND ""personnel""[All Fields]) OR ""research personnel""[All Fields] OR ""researcher""[All Fields] OR ""researchers""[All Fields] OR ""research""[MeSH Terms] OR ""research""[All Fields] OR ""research s""[All Fields] OR ""researchable""[All Fields] OR ""researche""[All Fields] OR ""researched""[All Fields] OR ""researcher s""[All Fields] OR ""researches""[All Fields] OR ""researching""[All Fields] OR ""researchs""[All Fields]) OR (""clinical trial""[Publication Type] OR ""clinical trials as topic""[MeSH Terms] OR ""clinical trial""[All Fields]) OR (""randomized controlled trial""[Publication Type] OR ""randomized controlled trials as topic""[MeSH Terms] OR ""randomized controlled trial""[All Fields] OR ""randomised controlled trial""[All Fields]))",Most Recent,,"""studies""[All Fields] OR ""study""[All Fields] OR ""study s""[All Fields] OR ""studying""[All Fields] OR ""studys""[All Fields] OR (""research personnel""[MeSH Terms] OR (""research""[All Fields] AND ""personnel""[All Fields]) OR ""research personnel""[All Fields] OR ""researcher""[All Fields] OR ""researchers""[All Fields] OR ""research""[MeSH Terms] OR ""research""[All Fields] OR ""research s""[All Fields] OR ""researchable""[All Fields] OR ""researche""[All Fields] OR ""researched""[All Fields] OR ""researcher s""[All Fields] OR ""researches""[All Fields] OR ""researching""[All Fields] OR ""researchs""[All Fields]) OR (""clinical trial""[Publication Type] OR ""clinical trials as topic""[MeSH Terms] OR ""clinical trial""[All Fields]) OR (""randomized controlled trial""[Publication Type] OR ""randomized controlled trials as topic""[MeSH Terms] OR ""randomized controlled trial""[All Fields] OR ""randomised controlled trial""[All Fields])" | 23,805,719 |
| 4 | "(""equities""[All Fields] OR ""equity""[All Fields] OR ""disparit*""[All Fields] OR ""inequit*""[All Fields] OR ""inequalit*""[All Fields])",Most Recent,,"""equities""[All Fields] OR ""equity""[All Fields] OR ""disparit*""[All Fields] OR ""inequit*""[All Fields] OR ""inequalit*""[All Fields]" | 273,910 |
| 3 | 3,"(""patient safety""[MeSH Terms] OR (""patient""[All Fields] AND ""safety""[All Fields]) OR ""patient safety""[All Fields] OR (""patient harm""[MeSH Terms] OR (""patient""[All Fields] AND ""harm""[All Fields]) OR ""patient harm""[All Fields]) OR ""safety management""[MeSH Terms])",Most Recent,,"""patient safety""[MeSH Terms] OR (""patient""[All Fields] AND ""safety""[All Fields]) OR ""patient safety""[All Fields] OR (""patient harm""[MeSH Terms] OR (""patient""[All Fields] AND ""harm""[All Fields]) OR ""patient harm""[All Fields]) OR ""safety management""[MeSH Terms]" | 253,755 |
| 2 | "(""effect""[All Fields] OR ""effecting""[All Fields] OR ""effective""[All Fields] OR ""effectively""[All Fields] OR ""effectiveness""[All Fields] OR ""effectivenesses""[All Fields] OR ""effectives""[All Fields] OR ""effectivities""[All Fields] OR ""effectivity""[All Fields] OR ""effects""[All Fields] OR ""effectiv*""[All Fields])",Most Recent,,"""effect""[All Fields] OR ""effecting""[All Fields] OR ""effective""[All Fields] OR ""effectively""[All Fields] OR ""effectiveness""[All Fields] OR ""effectivenesses""[All Fields] OR ""effectives""[All Fields] OR ""effectivities""[All Fields] OR ""effectivity""[All Fields] OR ""effects""[All Fields] OR ""effectiv*""[All Fields]" | 11,682,789 |
| 1 | edupression.com - Schema: all,Most Recent,,edupression.com | 0 |
| **Web of Science - Date Run: 30/07/2024 13:59:53** | | |
| ID | Search | Hits |
| 1 | ALL=(Edupression) | 0 |
| 2 | ALL=((effect OR effective OR effectively OR effectiveness OR effects OR effectiv*)) | 15644528 |
| 3 | ALL=((patient safety OR patient harm OR safety management)) | 682985 |
| 4 | ALL=((equities OR equity OR disparit* OR inequit* OR inequalit*)) | 636039 |
| 5 | ALL=((studies OR study OR studying OR studys OR research personnel OR research OR researcher OR researchers OR clinical trial OR clinical trials as topic OR clinical trial OR randomized controlled trial OR randomized controlled trials as topic OR randomized controlled trial OR randomised controlled trial)) | 38704259 |
| 6 | #1 AND #5 AND (#2 OR #3 OR #4) | 0 |
| **Web of Science - Date Run: 14/07/2025 13:30:57** | | |
| ID | Search | Hits |
| 1 | ALL=(Edupression) | 1 |
| 2 | ALL=(((effect OR effective OR effectively OR effectiveness OR effects OR effectiv*))) | 16777749 |
| 3 | ALL=((patient safety OR patient harm OR safety management)) | 758088 |
| 4 | ALL=((equities OR equity OR disparit* OR inequit* OR inequalit*)) | 713759 |
| 5 | ALL=((studies OR study OR studying OR studys OR research personnel OR research OR researcher OR researchers OR clinical trial OR clinical trials as topic OR clinical trial OR randomized controlled trial OR randomized controlled trials as topic OR randomized controlled trial OR randomised controlled trial)) | 41244021 |
| 6 | #1 AND #5 AND (#2 OR #3 OR #4) | 0 |

| **CENTRAL - Date Run: 14/07/2025 13:02:54** | | |
| --- | --- | --- |
| ID | Search | Hits |
| #1 | elona therapy Depression | 4 |
| #2 | effect OR effecting OR effective OR effectively OR effectiveness OR effectivenesses OR effectives OR effectivities OR effectivity OR effects OR effectiv | 1342628 |
| #3 | patient safety OR patient AND safety OR patient safety OR patient harm OR patient AND harm OR patient harm OR safety management | 128734 |
| #4 | equities OR equity OR disparit OR inequit OR inequalit | 2815 |
| #5 | studies OR study OR study s OR studying OR studys OR research personnel OR research AND personnel OR research personnel OR researcher OR researchers OR research OR research s OR researchable OR researche OR researched OR researchers OR researches OR researching OR researchs OR clinical trial OR clinical trials as topic OR clinical trial OR randomized controlled trial OR randomized controlled trials as topic OR randomized controlled trial OR randomised controlled trial | 1951451 |
| #6 | #1 AND #5 AND (#2 OR #3 OR #4) | 4 |
| **Pubmed - Date Run: 14/07/2025 09:40:28** | | |
| ID | Search | Hits |
| 6 | #1 AND #5 AND (#2 OR #3 OR #4),Most Recent,,"""edupression.com""[All Fields] AND (""studies""[All Fields] OR ""study""[All Fields] OR ""study s""[All Fields] OR ""studying""[All Fields] OR ""studys""[All Fields] OR (""research personnel""[MeSH Terms] OR (""research""[All Fields] AND ""personnel""[All Fields]) OR ""research personnel""[All Fields] OR ""researcher""[All Fields] OR ""researchers""[All Fields] OR ""research""[MeSH Terms] OR ""research""[All Fields] OR ""research s""[All Fields] OR ""researchable""[All Fields] OR ""researche""[All Fields] OR ""researched""[All Fields] OR ""researcher s""[All Fields] OR ""researches""[All Fields] OR ""researching""[All Fields] OR ""researchs""[All Fields]) OR (""clinical trial""[Publication Type] OR ""clinical trials as topic""[MeSH Terms] OR ""clinical trial""[All Fields]) OR (""randomized controlled trial""[Publication Type] OR ""randomized controlled trials as topic""[MeSH Terms] OR ""randomized controlled trial""[All Fields] OR ""randomised controlled trial""[All Fields])) AND (""effect""[All Fields] OR ""effecting""[All Fields] OR ""effective""[All Fields] OR ""effectively""[All Fields] OR ""effectiveness""[All Fields] OR ""effectivenesses""[All Fields] OR ""effectives""[All Fields] OR ""effectivities""[All Fields] OR ""effectivity""[All Fields] OR ""effects""[All Fields] OR ""effectiv*""[All Fields] OR (""patient safety""[MeSH Terms] OR (""patient""[All Fields] AND ""safety""[All Fields]) OR ""patient safety""[All Fields] OR (""patient harm""[MeSH Terms] OR (""patient""[All Fields] AND ""harm""[All Fields]) OR ""patient harm""[All Fields]) OR ""safety management""[MeSH Terms]) OR (""equities""[All Fields] OR ""equity""[All Fields] OR ""disparit*""[All Fields] OR ""inequit*""[All Fields] OR ""inequalit*""[All Fields]))" | 4 |
| 5 | "(""studies""[All Fields] OR ""study""[All Fields] OR ""study s""[All Fields] OR ""studying""[All Fields] OR ""studys""[All Fields] OR (""research personnel""[MeSH Terms] OR (""research""[All Fields] AND ""personnel""[All Fields]) OR ""research personnel""[All Fields] OR ""researcher""[All Fields] OR ""researchers""[All Fields] OR ""research""[MeSH Terms] OR ""research""[All Fields] OR ""research s""[All Fields] OR ""researchable""[All Fields] OR ""researche""[All Fields] OR ""researched""[All Fields] OR ""researcher s""[All Fields] OR ""researches""[All Fields] OR ""researching""[All Fields] OR ""researchs""[All Fields]) OR (""clinical trial""[Publication Type] OR ""clinical trials as topic""[MeSH Terms] OR ""clinical trial""[All Fields]) OR (""randomized controlled trial""[Publication Type] OR ""randomized controlled trials as topic""[MeSH Terms] OR ""randomized controlled trial""[All Fields] OR ""randomised controlled trial""[All Fields]))",Most Recent,,"""studies""[All Fields] OR ""study""[All Fields] OR ""study s""[All Fields] OR ""studying""[All Fields] OR ""studys""[All Fields] OR (""research personnel""[MeSH Terms] OR (""research""[All Fields] AND ""personnel""[All Fields]) OR ""research personnel""[All Fields] OR ""researcher""[All Fields] OR ""researchers""[All Fields] OR ""research""[MeSH Terms] OR ""research""[All Fields] OR ""research s""[All Fields] OR ""researchable""[All Fields] OR ""researche""[All Fields] OR ""researched""[All Fields] OR ""researcher s""[All Fields] OR ""researches""[All Fields] OR ""researching""[All Fields] OR ""researchs""[All Fields]) OR (""clinical trial""[Publication Type] OR ""clinical trials as topic""[MeSH Terms] OR ""clinical trial""[All Fields]) OR (""randomized controlled trial""[Publication Type] OR ""randomized controlled trials as topic""[MeSH Terms] OR ""randomized controlled trial""[All Fields] OR ""randomised controlled trial""[All Fields])" | 23,805,719 |
| 4 | "(""equities""[All Fields] OR ""equity""[All Fields] OR ""disparit*""[All Fields] OR ""inequit*""[All Fields] OR ""inequalit*""[All Fields])",Most Recent,,"""equities""[All Fields] OR ""equity""[All Fields] OR ""disparit*""[All Fields] OR ""inequit*""[All Fields] OR ""inequalit*""[All Fields]" | 273,910 |
| 3 | 3,"(""patient safety""[MeSH Terms] OR (""patient""[All Fields] AND ""safety""[All Fields]) OR ""patient safety""[All Fields] OR (""patient harm""[MeSH Terms] OR (""patient""[All Fields] AND ""harm""[All Fields]) OR ""patient harm""[All Fields]) OR ""safety management""[MeSH Terms])",Most Recent,,"""patient safety""[MeSH Terms] OR (""patient""[All Fields] AND ""safety""[All Fields]) OR ""patient safety""[All Fields] OR (""patient harm""[MeSH Terms] OR (""patient""[All Fields] AND ""harm""[All Fields]) OR ""patient harm""[All Fields]) OR ""safety management""[MeSH Terms]" | 253,755 |
| 2 | "(""effect""[All Fields] OR ""effecting""[All Fields] OR ""effective""[All Fields] OR ""effectively""[All Fields] OR ""effectiveness""[All Fields] OR ""effectivenesses""[All Fields] OR ""effectives""[All Fields] OR ""effectivities""[All Fields] OR ""effectivity""[All Fields] OR ""effects""[All Fields] OR ""effectiv*""[All Fields])",Most Recent,,"""effect""[All Fields] OR ""effecting""[All Fields] OR ""effective""[All Fields] OR ""effectively""[All Fields] OR ""effectiveness""[All Fields] OR ""effectivenesses""[All Fields] OR ""effectives""[All Fields] OR ""effectivities""[All Fields] OR ""effectivity""[All Fields] OR ""effects""[All Fields] OR ""effectiv*""[All Fields]" | 11,682,789 |
| 1 | elona therapy depression,Most Recent,,""""""elona""""[All Fields] AND (""""therapeutics""""[MeSH Terms] OR """"therapeutics""""[All Fields] OR """"therapies""""[All Fields] OR """"therapy""""[MeSH Subheading] OR """"therapy""""[All Fields] OR """"therapy s""""[All Fields] OR """"therapys""""[All Fields]) AND (""""depressed""""[All Fields] OR """"depression""""[MeSH Terms] OR """"depression""""[All Fields] OR """"depressions""""[All Fields] OR """"depression s""""[All Fields] OR """"depressive disorder""""[MeSH Terms] OR (""""depressive""""[All Fields] AND """"disorder""""[All Fields]) OR """"depressive disorder""""[All Fields] OR """"depressivity""""[All Fields] OR """"depressive""""[All Fields] OR """"depressively""""[All Fields] OR """"depressiveness""""[All Fields] OR """"depressives""""[All Fields])"" | 8 |
| **Web of Science - Date Run: 14/07/2025 13:26:31** | | |
| ID | Search | Hits |
| 1 | ALL=(elona therapy depression) | 7 |
| 2 | ALL=(((effect OR effective OR effectively OR effectiveness OR effects OR effectiv*))) | 16777749 |
| 3 | ALL=((patient safety OR patient harm OR safety management)) | 758088 |
| 4 | ALL=((equities OR equity OR disparit* OR inequit* OR inequalit*)) | 713759 |
| 5 | ALL=((studies OR study OR studying OR studys OR research personnel OR research OR researcher OR researchers OR clinical trial OR clinical trials as topic OR clinical trial OR randomized controlled trial OR randomized controlled trials as topic OR randomized controlled trial OR randomised controlled trial)) | 41244021 |
| 6 | #1 AND #5 AND (#2 OR #3 OR #4) | 4 |

| **CENTRAL - Date Run: 14/07/2025 13:11:54** | | |
| --- | --- | --- |
| ID | Search | Hits |
| #1 | my7step | 0 |
| #2 | effect OR effecting OR effective OR effectively OR effectiveness OR effectivenesses OR effectives OR effectivities OR effectivity OR effects OR effectiv | 1342628 |
| #3 | patient safety OR patient AND safety OR patient safety OR patient harm OR patient AND harm OR patient harm OR safety management | 128734 |
| #4 | equities OR equity OR disparit OR inequit OR inequalit | 2815 |
| #5 | studies OR study OR study s OR studying OR studys OR research personnel OR research AND personnel OR research personnel OR researcher OR researchers OR research OR research s OR researchable OR researche OR researched OR researchers OR researches OR researching OR researchs OR clinical trial OR clinical trials as topic OR clinical trial OR randomized controlled trial OR randomized controlled trials as topic OR randomized controlled trial OR randomised controlled trial | 1951451 |
| #6 | #1 AND #5 AND (#2 OR #3 OR #4) | 0 |
| **Pubmed - Date Run: 14/07/2025 09:40:28** | | |
| ID | Search | Hits |
| 6 | #1 AND #5 AND (#2 OR #3 OR #4),Most Recent,,"""edupression.com""[All Fields] AND (""studies""[All Fields] OR ""study""[All Fields] OR ""study s""[All Fields] OR ""studying""[All Fields] OR ""studys""[All Fields] OR (""research personnel""[MeSH Terms] OR (""research""[All Fields] AND ""personnel""[All Fields]) OR ""research personnel""[All Fields] OR ""researcher""[All Fields] OR ""researchers""[All Fields] OR ""research""[MeSH Terms] OR ""research""[All Fields] OR ""research s""[All Fields] OR ""researchable""[All Fields] OR ""researche""[All Fields] OR ""researched""[All Fields] OR ""researcher s""[All Fields] OR ""researches""[All Fields] OR ""researching""[All Fields] OR ""researchs""[All Fields]) OR (""clinical trial""[Publication Type] OR ""clinical trials as topic""[MeSH Terms] OR ""clinical trial""[All Fields]) OR (""randomized controlled trial""[Publication Type] OR ""randomized controlled trials as topic""[MeSH Terms] OR ""randomized controlled trial""[All Fields] OR ""randomised controlled trial""[All Fields])) AND (""effect""[All Fields] OR ""effecting""[All Fields] OR ""effective""[All Fields] OR ""effectively""[All Fields] OR ""effectiveness""[All Fields] OR ""effectivenesses""[All Fields] OR ""effectives""[All Fields] OR ""effectivities""[All Fields] OR ""effectivity""[All Fields] OR ""effects""[All Fields] OR ""effectiv*""[All Fields] OR (""patient safety""[MeSH Terms] OR (""patient""[All Fields] AND ""safety""[All Fields]) OR ""patient safety""[All Fields] OR (""patient harm""[MeSH Terms] OR (""patient""[All Fields] AND ""harm""[All Fields]) OR ""patient harm""[All Fields]) OR ""safety management""[MeSH Terms]) OR (""equities""[All Fields] OR ""equity""[All Fields] OR ""disparit*""[All Fields] OR ""inequit*""[All Fields] OR ""inequalit*""[All Fields]))" | 0 |
| 5 | "(""studies""[All Fields] OR ""study""[All Fields] OR ""study s""[All Fields] OR ""studying""[All Fields] OR ""studys""[All Fields] OR (""research personnel""[MeSH Terms] OR (""research""[All Fields] AND ""personnel""[All Fields]) OR ""research personnel""[All Fields] OR ""researcher""[All Fields] OR ""researchers""[All Fields] OR ""research""[MeSH Terms] OR ""research""[All Fields] OR ""research s""[All Fields] OR ""researchable""[All Fields] OR ""researche""[All Fields] OR ""researched""[All Fields] OR ""researcher s""[All Fields] OR ""researches""[All Fields] OR ""researching""[All Fields] OR ""researchs""[All Fields]) OR (""clinical trial""[Publication Type] OR ""clinical trials as topic""[MeSH Terms] OR ""clinical trial""[All Fields]) OR (""randomized controlled trial""[Publication Type] OR ""randomized controlled trials as topic""[MeSH Terms] OR ""randomized controlled trial""[All Fields] OR ""randomised controlled trial""[All Fields]))",Most Recent,,"""studies""[All Fields] OR ""study""[All Fields] OR ""study s""[All Fields] OR ""studying""[All Fields] OR ""studys""[All Fields] OR (""research personnel""[MeSH Terms] OR (""research""[All Fields] AND ""personnel""[All Fields]) OR ""research personnel""[All Fields] OR ""researcher""[All Fields] OR ""researchers""[All Fields] OR ""research""[MeSH Terms] OR ""research""[All Fields] OR ""research s""[All Fields] OR ""researchable""[All Fields] OR ""researche""[All Fields] OR ""researched""[All Fields] OR ""researcher s""[All Fields] OR ""researches""[All Fields] OR ""researching""[All Fields] OR ""researchs""[All Fields]) OR (""clinical trial""[Publication Type] OR ""clinical trials as topic""[MeSH Terms] OR ""clinical trial""[All Fields]) OR (""randomized controlled trial""[Publication Type] OR ""randomized controlled trials as topic""[MeSH Terms] OR ""randomized controlled trial""[All Fields] OR ""randomised controlled trial""[All Fields])" | 23,805,719 |
| 4 | "(""equities""[All Fields] OR ""equity""[All Fields] OR ""disparit*""[All Fields] OR ""inequit*""[All Fields] OR ""inequalit*""[All Fields])",Most Recent,,"""equities""[All Fields] OR ""equity""[All Fields] OR ""disparit*""[All Fields] OR ""inequit*""[All Fields] OR ""inequalit*""[All Fields]" | 317,276 |
| 3 | 3,"(""patient safety""[MeSH Terms] OR (""patient""[All Fields] AND ""safety""[All Fields]) OR ""patient safety""[All Fields] OR (""patient harm""[MeSH Terms] OR (""patient""[All Fields] AND ""harm""[All Fields]) OR ""patient harm""[All Fields]) OR ""safety management""[MeSH Terms])",Most Recent,,"""patient safety""[MeSH Terms] OR (""patient""[All Fields] AND ""safety""[All Fields]) OR ""patient safety""[All Fields] OR (""patient harm""[MeSH Terms] OR (""patient""[All Fields] AND ""harm""[All Fields]) OR ""patient harm""[All Fields]) OR ""safety management""[MeSH Terms]" | 278,587 |
| 2 | "(""effect""[All Fields] OR ""effecting""[All Fields] OR ""effective""[All Fields] OR ""effectively""[All Fields] OR ""effectiveness""[All Fields] OR ""effectivenesses""[All Fields] OR ""effectives""[All Fields] OR ""effectivities""[All Fields] OR ""effectivity""[All Fields] OR ""effects""[All Fields] OR ""effectiv*""[All Fields])",Most Recent,,"""effect""[All Fields] OR ""effecting""[All Fields] OR ""effective""[All Fields] OR ""effectively""[All Fields] OR ""effectiveness""[All Fields] OR ""effectivenesses""[All Fields] OR ""effectives""[All Fields] OR ""effectivities""[All Fields] OR ""effectivity""[All Fields] OR ""effects""[All Fields] OR ""effectiv*""[All Fields]" | 12,315,133 |
| 1 | My7steps - Schema: all,Most Recent,,My7steps, | 0 |
| **Web of Science - Date Run: 14/07/2025 13:30:57** | | |
| ID | Search | Hits |
| 1 | ALL=(My7step) | 0 |
| 2 | ALL=(((effect OR effective OR effectively OR effectiveness OR effects OR effectiv*))) | 16777749 |
| 3 | ALL=((patient safety OR patient harm OR safety management)) | 758088 |
| 4 | ALL=((equities OR equity OR disparit* OR inequit* OR inequalit*)) | 713759 |
| 5 | ALL=((studies OR study OR studying OR studys OR research personnel OR research OR researcher OR researchers OR clinical trial OR clinical trials as topic OR clinical trial OR randomized controlled trial OR randomized controlled trials as topic OR randomized controlled trial OR randomised controlled trial)) | 41244021 |
| 6 | #1 AND #5 AND (#2 OR #3 OR #4) | 0 |

| **CENTRAL - Date Run: 30/07/2024 22:18:57** | | |
| --- | --- | --- |
| ID | Search | Hits |
| #1 | novego | 8 |
| #2 | effect OR effecting OR effective OR effectively OR effectiveness OR effectivenesses OR effectives OR effectivities OR effectivity OR effects OR effectiv | 1288807 |
| #3 | patient safety OR patient AND safety OR patient safety OR patient harm OR patient AND harm OR patient harm OR safety management | 123223 |
| #4 | equities OR equity OR disparit OR inequit OR inequalit | 2251 |
| #5 | studies OR study OR study s OR studying OR studys OR research personnel OR research AND personnel OR research personnel OR researcher OR researchers OR research OR research s OR researchable OR researche OR researched OR researchers OR researches OR researching OR researchs OR clinical trial OR clinical trials as topic OR clinical trial OR randomized controlled trial OR randomized controlled trials as topic OR randomized controlled trial OR randomised controlled trial | 1865550 |
| **CENTRAL - Date Run: 14/07/2025 12:09:04** | | |
| ID | Search | Hits |
| #1 | Novego | 9 |
| #2 | effect OR effecting OR effective OR effectively OR effectiveness OR effectivenesses OR effectives OR effectivities OR effectivity OR effects OR effectiv | 1342628 |
| #3 | patient safety OR patient AND safety OR patient safety OR patient harm OR patient AND harm OR patient harm OR safety management | 128734 |
| #4 | equities OR equity OR disparit OR inequit OR inequalit | 2815 |
| #5 | studies OR study OR study s OR studying OR studys OR research personnel OR research AND personnel OR research personnel OR researcher OR researchers OR research OR research s OR researchable OR researche OR researched OR researchers OR researches OR researching OR researchs OR clinical trial OR clinical trials as topic OR clinical trial OR randomized controlled trial OR randomized controlled trials as topic OR randomized controlled trial OR randomised controlled trial | 1951451 |
| #6 | #1 AND #5 AND (#2 OR #3 OR #4) | 7 |
| **Pubmed - Date Run: 30/07/2024 09:37:27** | | |
| 7 | #2 AND #6 AND (#3 OR #4 OR #5),Most Recent,,"""novego""[All Fields] AND (""studies""[All Fields] OR ""study""[All Fields] OR ""study s""[All Fields] OR ""studying""[All Fields] OR ""studys""[All Fields] OR (""research personnel""[MeSH Terms] OR (""research""[All Fields] AND ""personnel""[All Fields]) OR ""research personnel""[All Fields] OR ""researcher""[All Fields] OR ""researchers""[All Fields] OR ""research""[MeSH Terms] OR ""research""[All Fields] OR ""research s""[All Fields] OR ""researchable""[All Fields] OR ""researche""[All Fields] OR ""researched""[All Fields] OR ""researcher s""[All Fields] OR ""researches""[All Fields] OR ""researching""[All Fields] OR ""researchs""[All Fields]) OR (""clinical trial""[Publication Type] OR ""clinical trials as topic""[MeSH Terms] OR ""clinical trial""[All Fields]) OR (""randomized controlled trial""[Publication Type] OR ""randomized controlled trials as topic""[MeSH Terms] OR ""randomized controlled trial""[All Fields] OR ""randomised controlled trial""[All Fields])) AND (""effect""[All Fields] OR ""effecting""[All Fields] OR ""effective""[All Fields] OR ""effectively""[All Fields] OR ""effectiveness""[All Fields] OR ""effectivenesses""[All Fields] OR ""effectives""[All Fields] OR ""effectivities""[All Fields] OR ""effectivity""[All Fields] OR ""effects""[All Fields] OR ""effectiv*""[All Fields] OR (""patient safety""[MeSH Terms] OR (""patient""[All Fields] AND ""safety""[All Fields]) OR ""patient safety""[All Fields] OR (""patient harm""[MeSH Terms] OR (""patient""[All Fields] AND ""harm""[All Fields]) OR ""patient harm""[All Fields]) OR ""safety management""[MeSH Terms]) OR (""equities""[All Fields] OR ""equity""[All Fields] OR ""disparit*""[All Fields] OR ""inequit*""[All Fields] OR ""inequalit*""[All Fields]))" | 2 |
| 6 | "(""studies""[All Fields] OR ""study""[All Fields] OR ""study s""[All Fields] OR ""studying""[All Fields] OR ""studys""[All Fields] OR (""research personnel""[MeSH Terms] OR (""research""[All Fields] AND ""personnel""[All Fields]) OR ""research personnel""[All Fields] OR ""researcher""[All Fields] OR ""researchers""[All Fields] OR ""research""[MeSH Terms] OR ""research""[All Fields] OR ""research s""[All Fields] OR ""researchable""[All Fields] OR ""researche""[All Fields] OR ""researched""[All Fields] OR ""researcher s""[All Fields] OR ""researches""[All Fields] OR ""researching""[All Fields] OR ""researchs""[All Fields]) OR (""clinical trial""[Publication Type] OR ""clinical trials as topic""[MeSH Terms] OR ""clinical trial""[All Fields]) OR (""randomized controlled trial""[Publication Type] OR ""randomized controlled trials as topic""[MeSH Terms] OR ""randomized controlled trial""[All Fields] OR ""randomised controlled trial""[All Fields]))",Most Recent,,"""studies""[All Fields] OR ""study""[All Fields] OR ""study s""[All Fields] OR ""studying""[All Fields] OR ""studys""[All Fields] OR (""research personnel""[MeSH Terms] OR (""research""[All Fields] AND ""personnel""[All Fields]) OR ""research personnel""[All Fields] OR ""researcher""[All Fields] OR ""researchers""[All Fields] OR ""research""[MeSH Terms] OR ""research""[All Fields] OR ""research s""[All Fields] OR ""researchable""[All Fields] OR ""researche""[All Fields] OR ""researched""[All Fields] OR ""researcher s""[All Fields] OR ""researches""[All Fields] OR ""researching""[All Fields] OR ""researchs""[All Fields]) OR (""clinical trial""[Publication Type] OR ""clinical trials as topic""[MeSH Terms] OR ""clinical trial""[All Fields]) OR (""randomized controlled trial""[Publication Type] OR ""randomized controlled trials as topic""[MeSH Terms] OR ""randomized controlled trial""[All Fields] OR ""randomised controlled trial""[All Fields])" | 22,576,098 |
| 5 | "(""equities""[All Fields] OR ""equity""[All Fields] OR ""disparit*""[All Fields] OR ""inequit*""[All Fields] OR ""inequalit*""[All Fields])",Most Recent,,"""equities""[All Fields] OR ""equity""[All Fields] OR ""disparit*""[All Fields] OR ""inequit*""[All Fields] OR ""inequalit*""[All Fields]" | 273,966 |
| 4 | "(""patient safety""[MeSH Terms] OR (""patient""[All Fields] AND ""safety""[All Fields]) OR ""patient safety""[All Fields] OR (""patient harm""[MeSH Terms] OR (""patient""[All Fields] AND ""harm""[All Fields]) OR ""patient harm""[All Fields]) OR ""safety management""[MeSH Terms])",Most Recent,,"""patient safety""[MeSH Terms] OR (""patient""[All Fields] AND ""safety""[All Fields]) OR ""patient safety""[All Fields] OR (""patient harm""[MeSH Terms] OR (""patient""[All Fields] AND ""harm""[All Fields]) OR ""patient harm""[All Fields]) OR ""safety management""[MeSH Terms]" | 253,795 |
| 3 | "(""effect""[All Fields] OR ""effecting""[All Fields] OR ""effective""[All Fields] OR ""effectively""[All Fields] OR ""effectiveness""[All Fields] OR ""effectivenesses""[All Fields] OR ""effectives""[All Fields] OR ""effectivities""[All Fields] OR ""effectivity""[All Fields] OR ""effects""[All Fields] OR ""effectiv*""[All Fields])",Most Recent,,"""effect""[All Fields] OR ""effecting""[All Fields] OR ""effective""[All Fields] OR ""effectively""[All Fields] OR ""effectiveness""[All Fields] OR ""effectivenesses""[All Fields] OR ""effectives""[All Fields] OR ""effectivities""[All Fields] OR ""effectivity""[All Fields] OR ""effects""[All Fields] OR ""effectiv*""[All Fields]" | 11,683,631 |
| 1 | novego - Spellcheck off,Most Recent,,"""novego""[All Fields]" | 2 |
| **Pubmed - Date Run: 14/07/2025 04:50:25** | | |
| ID | Search | Hits |
| 9 | #8 AND #5 AND (#2 OR #3 OR #4),Most Recent,,"""novego""[All Fields] AND (""studies""[All Fields] OR ""study""[All Fields] OR ""study s""[All Fields] OR ""studying""[All Fields] OR ""studys""[All Fields] OR (""research personnel""[MeSH Terms] OR (""research""[All Fields] AND ""personnel""[All Fields]) OR ""research personnel""[All Fields] OR ""researcher""[All Fields] OR ""researchers""[All Fields] OR ""research""[MeSH Terms] OR ""research""[All Fields] OR ""research s""[All Fields] OR ""researchable""[All Fields] OR ""researche""[All Fields] OR ""researched""[All Fields] OR ""researcher s""[All Fields] OR ""researches""[All Fields] OR ""researching""[All Fields] OR ""researchs""[All Fields]) OR (""clinical trial""[Publication Type] OR ""clinical trials as topic""[MeSH Terms] OR ""clinical trial""[All Fields]) OR (""randomized controlled trial""[Publication Type] OR ""randomized controlled trials as topic""[MeSH Terms] OR ""randomized controlled trial""[All Fields] OR ""randomised controlled trial""[All Fields])) AND (""effect""[All Fields] OR ""effecting""[All Fields] OR ""effective""[All Fields] OR ""effectively""[All Fields] OR ""effectiveness""[All Fields] OR ""effectivenesses""[All Fields] OR ""effectives""[All Fields] OR ""effectivities""[All Fields] OR ""effectivity""[All Fields] OR ""effects""[All Fields] OR ""effectiv*""[All Fields] OR (""patient safety""[MeSH Terms] OR (""patient""[All Fields] AND ""safety""[All Fields]) OR ""patient safety""[All Fields] OR (""patient harm""[MeSH Terms] OR (""patient""[All Fields] AND ""harm""[All Fields]) OR ""patient harm""[All Fields]) OR ""safety management""[MeSH Terms]) OR (""equities""[All Fields] OR ""equity""[All Fields] OR ""disparit*""[All Fields] OR ""inequit*""[All Fields] OR ""inequalit*""[All Fields]))" | 2 |
| 8 | novego - Spellcheck off,Most Recent,,"""novego""[All Fields]" | 2 |
| 5 | "(""studies""[All Fields] OR ""study""[All Fields] OR ""study s""[All Fields] OR ""studying""[All Fields] OR ""studys""[All Fields] OR (""research personnel""[MeSH Terms] OR (""research""[All Fields] AND ""personnel""[All Fields]) OR ""research personnel""[All Fields] OR ""researcher""[All Fields] OR ""researchers""[All Fields] OR ""research""[MeSH Terms] OR ""research""[All Fields] OR ""research s""[All Fields] OR ""researchable""[All Fields] OR ""researche""[All Fields] OR ""researched""[All Fields] OR ""researcher s""[All Fields] OR ""researches""[All Fields] OR ""researching""[All Fields] OR ""researchs""[All Fields]) OR (""clinical trial""[Publication Type] OR ""clinical trials as topic""[MeSH Terms] OR ""clinical trial""[All Fields]) OR (""randomized controlled trial""[Publication Type] OR ""randomized controlled trials as topic""[MeSH Terms] OR ""randomized controlled trial""[All Fields] OR ""randomised controlled trial""[All Fields])",Most Recent,,"""studies""[All Fields] OR ""study""[All Fields] OR ""study s""[All Fields] OR ""studying""[All Fields] OR ""studys""[All Fields] OR (""research personnel""[MeSH Terms] OR (""research""[All Fields] AND ""personnel""[All Fields]) OR ""research personnel""[All Fields] OR ""researcher""[All Fields] OR ""researchers""[All Fields] OR ""research""[MeSH Terms] OR ""research""[All Fields] OR ""research s""[All Fields] OR ""researchable""[All Fields] OR ""researche""[All Fields] OR ""researched""[All Fields] OR ""researcher s""[All Fields] OR ""researches""[All Fields] OR ""researching""[All Fields] OR ""researchs""[All Fields]) OR (""clinical trial""[Publication Type] OR ""clinical trials as topic""[MeSH Terms] OR ""clinical trial""[All Fields]) OR (""randomized controlled trial""[Publication Type] OR ""randomized controlled trials as topic""[MeSH Terms] OR ""randomized controlled trial""[All Fields] OR ""randomised controlled trial""[All Fields])" | 23,805,719 |
| 4 | "(""equities""[All Fields] OR ""equity""[All Fields] OR ""disparit*""[All Fields] OR ""inequit*""[All Fields] OR ""inequalit*""[All Fields])",Most Recent,,"""equities""[All Fields] OR ""equity""[All Fields] OR ""disparit*""[All Fields] OR ""inequit*""[All Fields] OR ""inequalit*""[All Fields]" | 317,276 |
| 3 | (""patient safety""[MeSH Terms] OR (""patient""[All Fields] AND ""safety""[All Fields]) OR ""patient safety""[All Fields] OR (""patient harm""[MeSH Terms] OR (""patient""[All Fields] AND ""harm""[All Fields]) OR ""patient harm""[All Fields]) OR ""safety management""[MeSH Terms])",Most Recent,,"""patient safety""[MeSH Terms] OR (""patient""[All Fields] AND ""safety""[All Fields]) OR ""patient safety""[All Fields] OR (""patient harm""[MeSH Terms] OR (""patient""[All Fields] AND ""harm""[All Fields]) OR ""patient harm""[All Fields]) OR ""safety management""[MeSH Terms]" | 278,587 |
| 2 | "(""effect""[All Fields] OR ""effecting""[All Fields] OR ""effective""[All Fields] OR ""effectively""[All Fields] OR ""effectiveness""[All Fields] OR ""effectivenesses""[All Fields] OR ""effectives""[All Fields] OR ""effectivities""[All Fields] OR ""effectivity""[All Fields] OR ""effects""[All Fields] OR ""effectiv*""[All Fields])",Most Recent,,"""effect""[All Fields] OR ""effecting""[All Fields] OR ""effective""[All Fields] OR ""effectively""[All Fields] OR ""effectiveness""[All Fields] OR ""effectivenesses""[All Fields] OR ""effectives""[All Fields] OR ""effectivities""[All Fields] OR ""effectivity""[All Fields] OR ""effects""[All Fields] OR ""effectiv*""[All Fields]" | 12,315,133 |
| **Web of Science - Date Run: 30/07/2024 13:53:50** | | |
| ID | Search | Hits |
| 1 | ALL=(novego) | 3 |
| 2 | ALL=((effect OR effective OR effectively OR effectiveness OR effects OR effectiv*)) | 15644528 |
| 3 | ALL=((patient safety OR patient harm OR safety management)) | 682985 |
| 4 | ALL=((equities OR equity OR disparit* OR inequit* OR inequalit*)) | 636039 |
| 5 | ALL=((studies OR study OR studying OR studys OR research personnel OR research OR researcher OR researchers OR clinical trial OR clinical trials as topic OR clinical trial OR randomized controlled trial OR randomized controlled trials as topic OR randomized controlled trial OR randomised controlled trial)) | 38704259 |
| 6 | #1 AND #5 AND (#2 OR #3 OR #4) | 3 |
| **Web of Science - Date Run: 14/07/2025 13:37:03** | | |
| ID | Search | Hits |
| 1 | ALL=(novego) | 3 |
| 2 | ALL=(((effect OR effective OR effectively OR effectiveness OR effects OR effectiv*))) | 16777749 |
| 3 | ALL=((patient safety OR patient harm OR safety management)) | 758088 |
| 4 | ALL=((equities OR equity OR disparit* OR inequit* OR inequalit*)) | 713759 |
| 5 | ALL=((studies OR study OR studying OR studys OR research personnel OR research OR researcher OR researchers OR clinical trial OR clinical trials as topic OR clinical trial OR randomized controlled trial OR randomized controlled trials as topic OR randomized controlled trial OR randomised controlled trial)) | 41244021 |
| 6 | #1 AND #5 AND (#2 OR #3 OR #4) | 3 |

| **CENTRAL - Date Run: 30/07/2024 22:21:54** | | |
| --- | --- | --- |
| ID | Search | Hits |
| #1 | selfapy | 8 |
| #2 | effect OR effecting OR effective OR effectively OR effectiveness OR effectivenesses OR effectives OR effectivities OR effectivity OR effects OR effectiv | 1288807 |
| #3 | patient safety OR patient AND safety OR patient safety OR patient harm OR patient AND harm OR patient harm OR safety management | 123223 |
| #4 | equities OR equity OR disparit OR inequit OR inequalit | 2251 |
| #5 | studies OR study OR study s OR studying OR studys OR research personnel OR research AND personnel OR research personnel OR researcher OR researchers OR research OR research s OR researchable OR researche OR researched OR researchers OR researches OR researching OR researchs OR clinical trial OR clinical trials as topic OR clinical trial OR randomized controlled trial OR randomized controlled trials as topic OR randomized controlled trial OR randomised controlled trial | 1865550 |
| #6 | #1 AND #5 AND (#2 OR #3 OR #4) | 7 |
| **CENTRAL - Date Run: 14/07/2025 12:16:23** | | |
| ID | Search | Hits |
| #1 | selfapy | 13 |
| #2 | effect OR effecting OR effective OR effectively OR effectiveness OR effectivenesses OR effectives OR effectivities OR effectivity OR effects OR effectiv | 1342628 |
| #3 | patient safety OR patient AND safety OR patient safety OR patient harm OR patient AND harm OR patient harm OR safety management | 128734 |
| #4 | equities OR equity OR disparit OR inequit OR inequalit | 2815 |
| #5 | studies OR study OR study s OR studying OR studys OR research personnel OR research AND personnel OR research personnel OR researcher OR researchers OR research OR research s OR researchable OR researche OR researched OR researchers OR researches OR researching OR researchs OR clinical trial OR clinical trials as topic OR clinical trial OR randomized controlled trial OR randomized controlled trials as topic OR randomized controlled trial OR randomised controlled trial | 1951451 |
| #6 | #1 AND #5 AND (#2 OR #3 OR #4) | 8 |
| **Pubmed - Date Run: 30/07/2024 09:29:08** | | |
| 6 | #1 AND #5 AND (#2 OR #3 OR #4),Most Recent,,"""selfapy""[All Fields] AND (""studies""[All Fields] OR ""study""[All Fields] OR ""study s""[All Fields] OR ""studying""[All Fields] OR ""studys""[All Fields] OR (""research personnel""[MeSH Terms] OR (""research""[All Fields] AND ""personnel""[All Fields]) OR ""research personnel""[All Fields] OR ""researcher""[All Fields] OR ""researchers""[All Fields] OR ""research""[MeSH Terms] OR ""research""[All Fields] OR ""research s""[All Fields] OR ""researchable""[All Fields] OR ""researche""[All Fields] OR ""researched""[All Fields] OR ""researcher s""[All Fields] OR ""researches""[All Fields] OR ""researching""[All Fields] OR ""researchs""[All Fields]) OR (""clinical trial""[Publication Type] OR ""clinical trials as topic""[MeSH Terms] OR ""clinical trial""[All Fields]) OR (""randomized controlled trial""[Publication Type] OR ""randomized controlled trials as topic""[MeSH Terms] OR ""randomized controlled trial""[All Fields] OR ""randomised controlled trial""[All Fields])) AND (""effect""[All Fields] OR ""effecting""[All Fields] OR ""effective""[All Fields] OR ""effectively""[All Fields] OR ""effectiveness""[All Fields] OR ""effectivenesses""[All Fields] OR ""effectives""[All Fields] OR ""effectivities""[All Fields] OR ""effectivity""[All Fields] OR ""effects""[All Fields] OR ""effectiv*""[All Fields] OR (""patient safety""[MeSH Terms] OR (""patient""[All Fields] AND ""safety""[All Fields]) OR ""patient safety""[All Fields] OR (""patient harm""[MeSH Terms] OR (""patient""[All Fields] AND ""harm""[All Fields]) OR ""patient harm""[All Fields]) OR ""safety management""[MeSH Terms]) OR (""equities""[All Fields] OR ""equity""[All Fields] OR ""disparit*""[All Fields] OR ""inequit*""[All Fields] OR ""inequalit*""[All Fields]))" | 5 |
| 5 | "(""studies""[All Fields] OR ""study""[All Fields] OR ""study s""[All Fields] OR ""studying""[All Fields] OR ""studys""[All Fields] OR (""research personnel""[MeSH Terms] OR (""research""[All Fields] AND ""personnel""[All Fields]) OR ""research personnel""[All Fields] OR ""researcher""[All Fields] OR ""researchers""[All Fields] OR ""research""[MeSH Terms] OR ""research""[All Fields] OR ""research s""[All Fields] OR ""researchable""[All Fields] OR ""researche""[All Fields] OR ""researched""[All Fields] OR ""researcher s""[All Fields] OR ""researches""[All Fields] OR ""researching""[All Fields] OR ""researchs""[All Fields]) OR (""clinical trial""[Publication Type] OR ""clinical trials as topic""[MeSH Terms] OR ""clinical trial""[All Fields]) OR (""randomized controlled trial""[Publication Type] OR ""randomized controlled trials as topic""[MeSH Terms] OR ""randomized controlled trial""[All Fields] OR ""randomised controlled trial""[All Fields]))",Most Recent,,"""studies""[All Fields] OR ""study""[All Fields] OR ""study s""[All Fields] OR ""studying""[All Fields] OR ""studys""[All Fields] OR (""research personnel""[MeSH Terms] OR (""research""[All Fields] AND ""personnel""[All Fields]) OR ""research personnel""[All Fields] OR ""researcher""[All Fields] OR ""researchers""[All Fields] OR ""research""[MeSH Terms] OR ""research""[All Fields] OR ""research s""[All Fields] OR ""researchable""[All Fields] OR ""researche""[All Fields] OR ""researched""[All Fields] OR ""researcher s""[All Fields] OR ""researches""[All Fields] OR ""researching""[All Fields] OR ""researchs""[All Fields]) OR (""clinical trial""[Publication Type] OR ""clinical trials as topic""[MeSH Terms] OR ""clinical trial""[All Fields]) OR (""randomized controlled trial""[Publication Type] OR ""randomized controlled trials as topic""[MeSH Terms] OR ""randomized controlled trial""[All Fields] OR ""randomised controlled trial""[All Fields])" | 22,576,098 |
| 4 | "(""equities""[All Fields] OR ""equity""[All Fields] OR ""disparit*""[All Fields] OR ""inequit*""[All Fields] OR ""inequalit*""[All Fields])",Most Recent,,"""equities""[All Fields] OR ""equity""[All Fields] OR ""disparit*""[All Fields] OR ""inequit*""[All Fields] OR ""inequalit*""[All Fields]" | 273,966 |
| 3 | "(""patient safety""[MeSH Terms] OR (""patient""[All Fields] AND ""safety""[All Fields]) OR ""patient safety""[All Fields] OR (""patient harm""[MeSH Terms] OR (""patient""[All Fields] AND ""harm""[All Fields]) OR ""patient harm""[All Fields]) OR ""safety management""[MeSH Terms])",Most Recent,,"""patient safety""[MeSH Terms] OR (""patient""[All Fields] AND ""safety""[All Fields]) OR ""patient safety""[All Fields] OR (""patient harm""[MeSH Terms] OR (""patient""[All Fields] AND ""harm""[All Fields]) OR ""patient harm""[All Fields]) OR ""safety management""[MeSH Terms]" | 253,795 |
| 2 | "(""effect""[All Fields] OR ""effecting""[All Fields] OR ""effective""[All Fields] OR ""effectively""[All Fields] OR ""effectiveness""[All Fields] OR ""effectivenesses""[All Fields] OR ""effectives""[All Fields] OR ""effectivities""[All Fields] OR ""effectivity""[All Fields] OR ""effects""[All Fields] OR ""effectiv*""[All Fields])",Most Recent,,"""effect""[All Fields] OR ""effecting""[All Fields] OR ""effective""[All Fields] OR ""effectively""[All Fields] OR ""effectiveness""[All Fields] OR ""effectivenesses""[All Fields] OR ""effectives""[All Fields] OR ""effectivities""[All Fields] OR ""effectivity""[All Fields] OR ""effects""[All Fields] OR ""effectiv*""[All Fields]" | 11,683,631 |
| 1 | selfapy,Most Recent,,"""selfapy""[All Fields]" | 8 |
| **Pubmed - Date Run: 14/07/2025 04:50:25** | | |
| ID | Search | Hits |
| 11 | #10 AND #5 AND (#2 OR #3 OR #4),Most Recent,,"""selfapy""[All Fields] AND (""studies""[All Fields] OR ""study""[All Fields] OR ""study s""[All Fields] OR ""studying""[All Fields] OR ""studys""[All Fields] OR (""research personnel""[MeSH Terms] OR (""research""[All Fields] AND ""personnel""[All Fields]) OR ""research personnel""[All Fields] OR ""researcher""[All Fields] OR ""researchers""[All Fields] OR ""research""[MeSH Terms] OR ""research""[All Fields] OR ""research s""[All Fields] OR ""researchable""[All Fields] OR ""researche""[All Fields] OR ""researched""[All Fields] OR ""researcher s""[All Fields] OR ""researches""[All Fields] OR ""researching""[All Fields] OR ""researchs""[All Fields]) OR (""clinical trial""[Publication Type] OR ""clinical trials as topic""[MeSH Terms] OR ""clinical trial""[All Fields]) OR (""randomized controlled trial""[Publication Type] OR ""randomized controlled trials as topic""[MeSH Terms] OR ""randomized controlled trial""[All Fields] OR ""randomised controlled trial""[All Fields])) AND (""effect""[All Fields] OR ""effecting""[All Fields] OR ""effective""[All Fields] OR ""effectively""[All Fields] OR ""effectiveness""[All Fields] OR ""effectivenesses""[All Fields] OR ""effectives""[All Fields] OR ""effectivities""[All Fields] OR ""effectivity""[All Fields] OR ""effects""[All Fields] OR ""effectiv*""[All Fields] OR (""patient safety""[MeSH Terms] OR (""patient""[All Fields] AND ""safety""[All Fields]) OR ""patient safety""[All Fields] OR (""patient harm""[MeSH Terms] OR (""patient""[All Fields] AND ""harm""[All Fields]) OR ""patient harm""[All Fields]) OR ""safety management""[MeSH Terms]) OR (""equities""[All Fields] OR ""equity""[All Fields] OR ""disparit*""[All Fields] OR ""inequit*""[All Fields] OR ""inequalit*""[All Fields]))" | 6 |
| 10 | selfapy,Most Recent,,"""selfapy""[All Fields]" | 10 |
| 5 | "(""studies""[All Fields] OR ""study""[All Fields] OR ""study s""[All Fields] OR ""studying""[All Fields] OR ""studys""[All Fields] OR (""research personnel""[MeSH Terms] OR (""research""[All Fields] AND ""personnel""[All Fields]) OR ""research personnel""[All Fields] OR ""researcher""[All Fields] OR ""researchers""[All Fields] OR ""research""[MeSH Terms] OR ""research""[All Fields] OR ""research s""[All Fields] OR ""researchable""[All Fields] OR ""researche""[All Fields] OR ""researched""[All Fields] OR ""researcher s""[All Fields] OR ""researches""[All Fields] OR ""researching""[All Fields] OR ""researchs""[All Fields]) OR (""clinical trial""[Publication Type] OR ""clinical trials as topic""[MeSH Terms] OR ""clinical trial""[All Fields]) OR (""randomized controlled trial""[Publication Type] OR ""randomized controlled trials as topic""[MeSH Terms] OR ""randomized controlled trial""[All Fields] OR ""randomised controlled trial""[All Fields])",Most Recent,,"""studies""[All Fields] OR ""study""[All Fields] OR ""study s""[All Fields] OR ""studying""[All Fields] OR ""studys""[All Fields] OR (""research personnel""[MeSH Terms] OR (""research""[All Fields] AND ""personnel""[All Fields]) OR ""research personnel""[All Fields] OR ""researcher""[All Fields] OR ""researchers""[All Fields] OR ""research""[MeSH Terms] OR ""research""[All Fields] OR ""research s""[All Fields] OR ""researchable""[All Fields] OR ""researche""[All Fields] OR ""researched""[All Fields] OR ""researcher s""[All Fields] OR ""researches""[All Fields] OR ""researching""[All Fields] OR ""researchs""[All Fields]) OR (""clinical trial""[Publication Type] OR ""clinical trials as topic""[MeSH Terms] OR ""clinical trial""[All Fields]) OR (""randomized controlled trial""[Publication Type] OR ""randomized controlled trials as topic""[MeSH Terms] OR ""randomized controlled trial""[All Fields] OR ""randomised controlled trial""[All Fields])" | 23,805,719 |
| 4 | "(""equities""[All Fields] OR ""equity""[All Fields] OR ""disparit*""[All Fields] OR ""inequit*""[All Fields] OR ""inequalit*""[All Fields])",Most Recent,,"""equities""[All Fields] OR ""equity""[All Fields] OR ""disparit*""[All Fields] OR ""inequit*""[All Fields] OR ""inequalit*""[All Fields]" | 317,276 |
| 3 | (""patient safety""[MeSH Terms] OR (""patient""[All Fields] AND ""safety""[All Fields]) OR ""patient safety""[All Fields] OR (""patient harm""[MeSH Terms] OR (""patient""[All Fields] AND ""harm""[All Fields]) OR ""patient harm""[All Fields]) OR ""safety management""[MeSH Terms])",Most Recent,,"""patient safety""[MeSH Terms] OR (""patient""[All Fields] AND ""safety""[All Fields]) OR ""patient safety""[All Fields] OR (""patient harm""[MeSH Terms] OR (""patient""[All Fields] AND ""harm""[All Fields]) OR ""patient harm""[All Fields]) OR ""safety management""[MeSH Terms]" | 278,587 |
| 2 | "(""effect""[All Fields] OR ""effecting""[All Fields] OR ""effective""[All Fields] OR ""effectively""[All Fields] OR ""effectiveness""[All Fields] OR ""effectivenesses""[All Fields] OR ""effectives""[All Fields] OR ""effectivities""[All Fields] OR ""effectivity""[All Fields] OR ""effects""[All Fields] OR ""effectiv*""[All Fields])",Most Recent,,"""effect""[All Fields] OR ""effecting""[All Fields] OR ""effective""[All Fields] OR ""effectively""[All Fields] OR ""effectiveness""[All Fields] OR ""effectivenesses""[All Fields] OR ""effectives""[All Fields] OR ""effectivities""[All Fields] OR ""effectivity""[All Fields] OR ""effects""[All Fields] OR ""effectiv*""[All Fields]" | 12,315,133 |
| **Web of Science - Date Run: 30/07/2024 13:59:53** | | |
| ID | Search | Hits |
| 1 | ALL=(selfapy) | 12 |
| 2 | ALL=((effect OR effective OR effectively OR effectiveness OR effects OR effectiv*)) | 15644528 |
| 3 | ALL=((patient safety OR patient harm OR safety management)) | 682985 |
| 4 | ALL=((equities OR equity OR disparit* OR inequit* OR inequalit*)) | 636039 |
| 5 | ALL=((studies OR study OR studying OR studys OR research personnel OR research OR researcher OR researchers OR clinical trial OR clinical trials as topic OR clinical trial OR randomized controlled trial OR randomized controlled trials as topic OR randomized controlled trial OR randomised controlled trial)) | 38704259 |
| 6 | #1 AND #5 AND (#2 OR #3 OR #4) | 11 |
| **Web of Science - Date Run: 14/07/2025 13:37:03** | | |
| ID | Search | Hits |
| 1 | ALL=(selfapy) | 14 |
| 2 | ALL=(((effect OR effective OR effectively OR effectiveness OR effects OR effectiv*))) | 16777749 |
| 3 | ALL=((patient safety OR patient harm OR safety management)) | 758088 |
| 4 | ALL=((equities OR equity OR disparit* OR inequit* OR inequalit*)) | 713759 |
| 5 | ALL=((studies OR study OR studying OR studys OR research personnel OR research OR researcher OR researchers OR clinical trial OR clinical trials as topic OR clinical trial OR randomized controlled trial OR randomized controlled trials as topic OR randomized controlled trial OR randomised controlled trial)) | 41244021 |
| 6 | #1 AND #5 AND (#2 OR #3 OR #4) | 13 |

| **CENTRAL - Date Run: 30/07/2024 22:21:54** | | |
| --- | --- | --- |
| ID | Search | Hits |
| #1 | novego | 8 |
| #2 | effect OR effecting OR effective OR effectively OR effectiveness OR effectivenesses OR effectives OR effectivities OR effectivity OR effects OR effectiv | 1288807 |
| #3 | patient safety OR patient AND safety OR patient safety OR patient harm OR patient AND harm OR patient harm OR safety management | 123223 |
| #4 | equities OR equity OR disparit OR inequit OR inequalit | 2251 |
| #5 | studies OR study OR study s OR studying OR studys OR research personnel OR research AND personnel OR research personnel OR researcher OR researchers OR research OR research s OR researchable OR researche OR researched OR researchers OR researches OR researching OR researchs OR clinical trial OR clinical trials as topic OR clinical trial OR randomized controlled trial OR randomized controlled trials as topic OR randomized controlled trial OR randomised controlled trial | 1865550 |
| #6 | #1 AND #5 AND (#2 OR #3 OR #4) | 6 |
| **CENTRAL - Date Run: 14/07/2025 12:16:23** | | |
| ID | Search | Hits |
| #1 | novego | 9 |
| #2 | effect OR effecting OR effective OR effectively OR effectiveness OR effectivenesses OR effectives OR effectivities OR effectivity OR effects OR effectiv | 1342628 |
| #3 | patient safety OR patient AND safety OR patient safety OR patient harm OR patient AND harm OR patient harm OR safety management | 128734 |
| #4 | equities OR equity OR disparit OR inequit OR inequalit | 2815 |
| #5 | studies OR study OR study s OR studying OR studys OR research personnel OR research AND personnel OR research personnel OR researcher OR researchers OR research OR research s OR researchable OR researche OR researched OR researchers OR researches OR researching OR researchs OR clinical trial OR clinical trials as topic OR clinical trial OR randomized controlled trial OR randomized controlled trials as topic OR randomized controlled trial OR randomised controlled trial | 1951451 |
| #6 | #1 AND #5 AND (#2 OR #3 OR #4) | 7 |
| **Pubmed - Date Run: 30/07/2024 09:37:08** | | |
| 6 | #1 AND #5 AND (#2 OR #3 OR #4), Most Recent,,"""novego""[All Fields] AND (""studies""[All Fields] OR ""study""[All Fields] OR ""study s""[All Fields] OR ""studying""[All Fields] OR ""studys""[All Fields] OR (""research personnel""[MeSH Terms] OR (""research""[All Fields] AND ""personnel""[All Fields]) OR ""research personnel""[All Fields] OR ""researcher""[All Fields] OR ""researchers""[All Fields] OR ""research""[MeSH Terms] OR ""research""[All Fields] OR ""research s""[All Fields] OR ""researchable""[All Fields] OR ""researche""[All Fields] OR ""researched""[All Fields] OR ""researcher s""[All Fields] OR ""researches""[All Fields] OR ""researching""[All Fields] OR ""researchs""[All Fields]) OR (""clinical trial""[Publication Type] OR ""clinical trials as topic""[MeSH Terms] OR ""clinical trial""[All Fields]) OR (""randomized controlled trial""[Publication Type] OR ""randomized controlled trials as topic""[MeSH Terms] OR ""randomized controlled trial""[All Fields] OR ""randomised controlled trial""[All Fields])) AND (""effect""[All Fields] OR ""effecting""[All Fields] OR ""effective""[All Fields] OR ""effectively""[All Fields] OR ""effectiveness""[All Fields] OR ""effectivenesses""[All Fields] OR ""effectives""[All Fields] OR ""effectivities""[All Fields] OR ""effectivity""[All Fields] OR ""effects""[All Fields] OR ""effectiv*""[All Fields] OR (""patient safety""[MeSH Terms] OR (""patient""[All Fields] AND ""safety""[All Fields]) OR ""patient safety""[All Fields] OR (""patient harm""[MeSH Terms] OR (""patient""[All Fields] AND ""harm""[All Fields]) OR ""patient harm""[All Fields]) OR ""safety management""[MeSH Terms]) OR (""equities""[All Fields] OR ""equity""[All Fields] OR ""disparit*""[All Fields] OR ""inequit*""[All Fields] OR ""inequalit*""[All Fields]))" | 2 |
| 5 | "(""studies""[All Fields] OR ""study""[All Fields] OR ""study s""[All Fields] OR ""studying""[All Fields] OR ""studys""[All Fields] OR (""research personnel""[MeSH Terms] OR (""research""[All Fields] AND ""personnel""[All Fields]) OR ""research personnel""[All Fields] OR ""researcher""[All Fields] OR ""researchers""[All Fields] OR ""research""[MeSH Terms] OR ""research""[All Fields] OR ""research s""[All Fields] OR ""researchable""[All Fields] OR ""researche""[All Fields] OR ""researched""[All Fields] OR ""researcher s""[All Fields] OR ""researches""[All Fields] OR ""researching""[All Fields] OR ""researchs""[All Fields]) OR (""clinical trial""[Publication Type] OR ""clinical trials as topic""[MeSH Terms] OR ""clinical trial""[All Fields]) OR (""randomized controlled trial""[Publication Type] OR ""randomized controlled trials as topic""[MeSH Terms] OR ""randomized controlled trial""[All Fields] OR ""randomised controlled trial""[All Fields]))",Most Recent,,"""studies""[All Fields] OR ""study""[All Fields] OR ""study s""[All Fields] OR ""studying""[All Fields] OR ""studys""[All Fields] OR (""research personnel""[MeSH Terms] OR (""research""[All Fields] AND ""personnel""[All Fields]) OR ""research personnel""[All Fields] OR ""researcher""[All Fields] OR ""researchers""[All Fields] OR ""research""[MeSH Terms] OR ""research""[All Fields] OR ""research s""[All Fields] OR ""researchable""[All Fields] OR ""researche""[All Fields] OR ""researched""[All Fields] OR ""researcher s""[All Fields] OR ""researches""[All Fields] OR ""researching""[All Fields] OR ""researchs""[All Fields]) OR (""clinical trial""[Publication Type] OR ""clinical trials as topic""[MeSH Terms] OR ""clinical trial""[All Fields]) OR (""randomized controlled trial""[Publication Type] OR ""randomized controlled trials as topic""[MeSH Terms] OR ""randomized controlled trial""[All Fields] OR ""randomised controlled trial""[All Fields])" | 22,576,098 |
| 4 | "(""equities""[All Fields] OR ""equity""[All Fields] OR ""disparit*""[All Fields] OR ""inequit*""[All Fields] OR ""inequalit*""[All Fields])",Most Recent,,"""equities""[All Fields] OR ""equity""[All Fields] OR ""disparit*""[All Fields] OR ""inequit*""[All Fields] OR ""inequalit*""[All Fields]" | 273,966 |
| 3 | "(""patient safety""[MeSH Terms] OR (""patient""[All Fields] AND ""safety""[All Fields]) OR ""patient safety""[All Fields] OR (""patient harm""[MeSH Terms] OR (""patient""[All Fields] AND ""harm""[All Fields]) OR ""patient harm""[All Fields]) OR ""safety management""[MeSH Terms])",Most Recent,,"""patient safety""[MeSH Terms] OR (""patient""[All Fields] AND ""safety""[All Fields]) OR ""patient safety""[All Fields] OR (""patient harm""[MeSH Terms] OR (""patient""[All Fields] AND ""harm""[All Fields]) OR ""patient harm""[All Fields]) OR ""safety management""[MeSH Terms]" | 253,795 |
| 2 | "(""effect""[All Fields] OR ""effecting""[All Fields] OR ""effective""[All Fields] OR ""effectively""[All Fields] OR ""effectiveness""[All Fields] OR ""effectivenesses""[All Fields] OR ""effectives""[All Fields] OR ""effectivities""[All Fields] OR ""effectivity""[All Fields] OR ""effects""[All Fields] OR ""effectiv*""[All Fields])",Most Recent,,"""effect""[All Fields] OR ""effecting""[All Fields] OR ""effective""[All Fields] OR ""effectively""[All Fields] OR ""effectiveness""[All Fields] OR ""effectivenesses""[All Fields] OR ""effectives""[All Fields] OR ""effectivities""[All Fields] OR ""effectivity""[All Fields] OR ""effects""[All Fields] OR ""effectiv*""[All Fields]" | 11,683,631 |
| 1 | novego - Spellcheck off,Most Recent,,"""novego""[All Fields]" | 2 |
| **Pubmed - Date Run: 14/07/2025 03:55:49** | | |
| ID | Search | Hits |
| 6 | #1 AND #5 AND (#2 OR #3 OR #4), Most Recent,,"""novego""[All Fields] AND (""studies""[All Fields] OR ""study""[All Fields] OR ""study s""[All Fields] OR ""studying""[All Fields] OR ""studys""[All Fields] OR (""research personnel""[MeSH Terms] OR (""research""[All Fields] AND ""personnel""[All Fields]) OR ""research personnel""[All Fields] OR ""researcher""[All Fields] OR ""researchers""[All Fields] OR ""research""[MeSH Terms] OR ""research""[All Fields] OR ""research s""[All Fields] OR ""researchable""[All Fields] OR ""researche""[All Fields] OR ""researched""[All Fields] OR ""researcher s""[All Fields] OR ""researches""[All Fields] OR ""researching""[All Fields] OR ""researchs""[All Fields]) OR (""clinical trial""[Publication Type] OR ""clinical trials as topic""[MeSH Terms] OR ""clinical trial""[All Fields]) OR (""randomized controlled trial""[Publication Type] OR ""randomized controlled trials as topic""[MeSH Terms] OR ""randomized controlled trial""[All Fields] OR ""randomised controlled trial""[All Fields])) AND (""effect""[All Fields] OR ""effecting""[All Fields] OR ""effective""[All Fields] OR ""effectively""[All Fields] OR ""effectiveness""[All Fields] OR ""effectivenesses""[All Fields] OR ""effectives""[All Fields] OR ""effectivities""[All Fields] OR ""effectivity""[All Fields] OR ""effects""[All Fields] OR ""effectiv*""[All Fields] OR (""patient safety""[MeSH Terms] OR (""patient""[All Fields] AND ""safety""[All Fields]) OR ""patient safety""[All Fields] OR (""patient harm""[MeSH Terms] OR (""patient""[All Fields] AND ""harm""[All Fields]) OR ""patient harm""[All Fields]) OR ""safety management""[MeSH Terms]) OR (""equities""[All Fields] OR ""equity""[All Fields] OR ""disparit*""[All Fields] OR ""inequit*""[All Fields] OR ""inequalit*""[All Fields]))" | 2 |
| 5 | "(""studies""[All Fields] OR ""study""[All Fields] OR ""study s""[All Fields] OR ""studying""[All Fields] OR ""studys""[All Fields] OR (""research personnel""[MeSH Terms] OR (""research""[All Fields] AND ""personnel""[All Fields]) OR ""research personnel""[All Fields] OR ""researcher""[All Fields] OR ""researchers""[All Fields] OR ""research""[MeSH Terms] OR ""research""[All Fields] OR ""research s""[All Fields] OR ""researchable""[All Fields] OR ""researche""[All Fields] OR ""researched""[All Fields] OR ""researcher s""[All Fields] OR ""researches""[All Fields] OR ""researching""[All Fields] OR ""researchs""[All Fields]) OR (""clinical trial""[Publication Type] OR ""clinical trials as topic""[MeSH Terms] OR ""clinical trial""[All Fields]) OR (""randomized controlled trial""[Publication Type] OR ""randomized controlled trials as topic""[MeSH Terms] OR ""randomized controlled trial""[All Fields] OR ""randomised controlled trial""[All Fields]))",Most Recent,,"""studies""[All Fields] OR ""study""[All Fields] OR ""study s""[All Fields] OR ""studying""[All Fields] OR ""studys""[All Fields] OR (""research personnel""[MeSH Terms] OR (""research""[All Fields] AND ""personnel""[All Fields]) OR ""research personnel""[All Fields] OR ""researcher""[All Fields] OR ""researchers""[All Fields] OR ""research""[MeSH Terms] OR ""research""[All Fields] OR ""research s""[All Fields] OR ""researchable""[All Fields] OR ""researche""[All Fields] OR ""researched""[All Fields] OR ""researcher s""[All Fields] OR ""researches""[All Fields] OR ""researching""[All Fields] OR ""researchs""[All Fields]) OR (""clinical trial""[Publication Type] OR ""clinical trials as topic""[MeSH Terms] OR ""clinical trial""[All Fields]) OR (""randomized controlled trial""[Publication Type] OR ""randomized controlled trials as topic""[MeSH Terms] OR ""randomized controlled trial""[All Fields] OR ""randomised controlled trial""[All Fields])" | 23,805,719 |
| 4 | "(""equities""[All Fields] OR ""equity""[All Fields] OR ""disparit*""[All Fields] OR ""inequit*""[All Fields] OR ""inequalit*""[All Fields])",Most Recent,,"""equities""[All Fields] OR ""equity""[All Fields] OR ""disparit*""[All Fields] OR ""inequit*""[All Fields] OR ""inequalit*""[All Fields]" | 317,276 |
| 3 | "(""patient safety""[MeSH Terms] OR (""patient""[All Fields] AND ""safety""[All Fields]) OR ""patient safety""[All Fields] OR (""patient harm""[MeSH Terms] OR (""patient""[All Fields] AND ""harm""[All Fields]) OR ""patient harm""[All Fields]) OR ""safety management""[MeSH Terms])",Most Recent,,"""patient safety""[MeSH Terms] OR (""patient""[All Fields] AND ""safety""[All Fields]) OR ""patient safety""[All Fields] OR (""patient harm""[MeSH Terms] OR (""patient""[All Fields] AND ""harm""[All Fields]) OR ""patient harm""[All Fields]) OR ""safety management""[MeSH Terms]" | 278,587 |
| 2 | "(""effect""[All Fields] OR ""effecting""[All Fields] OR ""effective""[All Fields] OR ""effectively""[All Fields] OR ""effectiveness""[All Fields] OR ""effectivenesses""[All Fields] OR ""effectives""[All Fields] OR ""effectivities""[All Fields] OR ""effectivity""[All Fields] OR ""effects""[All Fields] OR ""effectiv*""[All Fields])",Most Recent,,"""effect""[All Fields] OR ""effecting""[All Fields] OR ""effective""[All Fields] OR ""effectively""[All Fields] OR ""effectiveness""[All Fields] OR ""effectivenesses""[All Fields] OR ""effectives""[All Fields] OR ""effectivities""[All Fields] OR ""effectivity""[All Fields] OR ""effects""[All Fields] OR ""effectiv*""[All Fields]" | 12,315,133 |
| 1 | novego - Spellcheck off,Most Recent,,"""novego""[All Fields]" | 2 |
| **Web of Science - Date Run: 30/07/2024 13:59:53** | | |
| ID | Search | Hits |
| 1 | ALL=(novego) | 3 |
| 2 | ALL=((effect OR effective OR effectively OR effectiveness OR effects OR effectiv*)) | 15644528 |
| 3 | ALL=((patient safety OR patient harm OR safety management)) | 682985 |
| 4 | ALL=((equities OR equity OR disparit* OR inequit* OR inequalit*)) | 636039 |
| 5 | ALL=((studies OR study OR studying OR studys OR research personnel OR research OR researcher OR researchers OR clinical trial OR clinical trials as topic OR clinical trial OR randomized controlled trial OR randomized controlled trials as topic OR randomized controlled trial OR randomised controlled trial)) | 38704259 |
| 6 | #1 AND #5 AND (#2 OR #3 OR #4) | 3 |
| **Web of Science - Date Run: 14/07/2025 13:37:03** | | |
| ID | Search | Hits |
| 1 | ALL=(novego) | 3 |
| 2 | ALL=(((effect OR effective OR effectively OR effectiveness OR effects OR effectiv*))) | 16777749 |
| 3 | ALL=((patient safety OR patient harm OR safety management)) | 758088 |
| 4 | ALL=((equities OR equity OR disparit* OR inequit* OR inequalit*)) | 713759 |
| 5 | ALL=((studies OR study OR studying OR studys OR research personnel OR research OR researcher OR researchers OR clinical trial OR clinical trials as topic OR clinical trial OR randomized controlled trial OR randomized controlled trials as topic OR randomized controlled trial OR randomised controlled trial)) | 41244021 |
| 6 | #1 AND #5 AND (#2 OR #3 OR #4) | 3 |

| **CENTRAL - Date Run: 30/07/2024 22:24:32** | | |
| --- | --- | --- |
| ID | Search | Hits |
| #1 | velibra | 3 |
| #2 | effect OR effecting OR effective OR effectively OR effectiveness OR effectivenesses OR effectives OR effectivities OR effectivity OR effects OR effectiv | 1288807 |
| #3 | patient safety OR patient AND safety OR patient safety OR patient harm OR patient AND harm OR patient harm OR safety management | 123223 |
| #4 | equities OR equity OR disparit OR inequit OR inequalit | 2251 |
| #5 | studies OR study OR study s OR studying OR studys OR research personnel OR research AND personnel OR research personnel OR researcher OR researchers OR research OR research s OR researchable OR researche OR researched OR researchers OR researches OR researching OR researchs OR clinical trial OR clinical trials as topic OR clinical trial OR randomized controlled trial OR randomized controlled trials as topic OR randomized controlled trial OR randomised controlled trial | 1865550 |
| #6 | #1 AND #5 AND (#2 OR #3 OR #4) | 3 |
| **CENTRAL - Date Run: 14/07/2025 12:21:15** | | |
| ID | Search | Hits |
| #1 | velibra | 3 |
| #2 | effect OR effecting OR effective OR effectively OR effectiveness OR effectivenesses OR effectives OR effectivities OR effectivity OR effects OR effectiv | 1342628 |
| #3 | patient safety OR patient AND safety OR patient safety OR patient harm OR patient AND harm OR patient harm OR safety management | 128734 |
| #4 | equities OR equity OR disparit OR inequit OR inequalit | 2815 |
| #5 | studies OR study OR study s OR studying OR studys OR research personnel OR research AND personnel OR research personnel OR researcher OR researchers OR research OR research s OR researchable OR researche OR researched OR researchers OR researches OR researching OR researchs OR clinical trial OR clinical trials as topic OR clinical trial OR randomized controlled trial OR randomized controlled trials as topic OR randomized controlled trial OR randomised controlled trial | 1951451 |
| #6 | #1 AND #5 AND (#2 OR #3 OR #4) | 3 |
| **Pubmed - Date Run: 14/07/2024 09:26:13** | | |
| ID | Search | Hits |
| 6 | #1 AND #5 AND (#2 OR #3 OR #4),Most Recent,,"""velibra""[All Fields] AND (""studies""[All Fields] OR ""study""[All Fields] OR ""study s""[All Fields] OR ""studying""[All Fields] OR ""studys""[All Fields] OR (""research personnel""[MeSH Terms] OR (""research""[All Fields] AND ""personnel""[All Fields]) OR ""research personnel""[All Fields] OR ""researcher""[All Fields] OR ""researchers""[All Fields] OR ""research""[MeSH Terms] OR ""research""[All Fields] OR ""research s""[All Fields] OR ""researchable""[All Fields] OR ""researche""[All Fields] OR ""researched""[All Fields] OR ""researcher s""[All Fields] OR ""researches""[All Fields] OR ""researching""[All Fields] OR ""researchs""[All Fields]) OR (""clinical trial""[Publication Type] OR ""clinical trials as topic""[MeSH Terms] OR ""clinical trial""[All Fields]) OR (""randomized controlled trial""[Publication Type] OR ""randomized controlled trials as topic""[MeSH Terms] OR ""randomized controlled trial""[All Fields] OR ""randomised controlled trial""[All Fields])) AND (""effect""[All Fields] OR ""effecting""[All Fields] OR ""effective""[All Fields] OR ""effectively""[All Fields] OR ""effectiveness""[All Fields] OR ""effectivenesses""[All Fields] OR ""effectives""[All Fields] OR ""effectivities""[All Fields] OR ""effectivity""[All Fields] OR ""effects""[All Fields] OR ""effectiv*""[All Fields] OR (""patient safety""[MeSH Terms] OR (""patient""[All Fields] AND ""safety""[All Fields]) OR ""patient safety""[All Fields] OR (""patient harm""[MeSH Terms] OR (""patient""[All Fields] AND ""harm""[All Fields]) OR ""patient harm""[All Fields]) OR ""safety management""[MeSH Terms]) OR (""equities""[All Fields] OR ""equity""[All Fields] OR ""disparit*""[All Fields] OR ""inequit*""[All Fields] OR ""inequalit*""[All Fields]))" | 1 |
| 5 | "(""studies""[All Fields] OR ""study""[All Fields] OR ""study s""[All Fields] OR ""studying""[All Fields] OR ""studys""[All Fields] OR (""research personnel""[MeSH Terms] OR (""research""[All Fields] AND ""personnel""[All Fields]) OR ""research personnel""[All Fields] OR ""researcher""[All Fields] OR ""researchers""[All Fields] OR ""research""[MeSH Terms] OR ""research""[All Fields] OR ""research s""[All Fields] OR ""researchable""[All Fields] OR ""researche""[All Fields] OR ""researched""[All Fields] OR ""researcher s""[All Fields] OR ""researches""[All Fields] OR ""researching""[All Fields] OR ""researchs""[All Fields]) OR (""clinical trial""[Publication Type] OR ""clinical trials as topic""[MeSH Terms] OR ""clinical trial""[All Fields]) OR (""randomized controlled trial""[Publication Type] OR ""randomized controlled trials as topic""[MeSH Terms] OR ""randomized controlled trial""[All Fields] OR ""randomised controlled trial""[All Fields])",Most Recent,,"""studies""[All Fields] OR ""study""[All Fields] OR ""study s""[All Fields] OR ""studying""[All Fields] OR ""studys""[All Fields] OR (""research personnel""[MeSH Terms] OR (""research""[All Fields] AND ""personnel""[All Fields]) OR ""research personnel""[All Fields] OR ""researcher""[All Fields] OR ""researchers""[All Fields] OR ""research""[MeSH Terms] OR ""research""[All Fields] OR ""research s""[All Fields] OR ""researchable""[All Fields] OR ""researche""[All Fields] OR ""researched""[All Fields] OR ""researcher s""[All Fields] OR ""researches""[All Fields] OR ""researching""[All Fields] OR ""researchs""[All Fields]) OR (""clinical trial""[Publication Type] OR ""clinical trials as topic""[MeSH Terms] OR ""clinical trial""[All Fields]) OR (""randomized controlled trial""[Publication Type] OR ""randomized controlled trials as topic""[MeSH Terms] OR ""randomized controlled trial""[All Fields] OR ""randomised controlled trial""[All Fields])" | 22,576,098 |
| 4 | "(""equities""[All Fields] OR ""equity""[All Fields] OR ""disparit*""[All Fields] OR ""inequit*""[All Fields] OR ""inequalit*""[All Fields])",Most Recent,,"""equities""[All Fields] OR ""equity""[All Fields] OR ""disparit*""[All Fields] OR ""inequit*""[All Fields] OR ""inequalit*""[All Fields]" | 273,966 |
| 3 | (""patient safety""[MeSH Terms] OR (""patient""[All Fields] AND ""safety""[All Fields]) OR ""patient safety""[All Fields] OR (""patient harm""[MeSH Terms] OR (""patient""[All Fields] AND ""harm""[All Fields]) OR ""patient harm""[All Fields]) OR ""safety management""[MeSH Terms])",Most Recent,,"""patient safety""[MeSH Terms] OR (""patient""[All Fields] AND ""safety""[All Fields]) OR ""patient safety""[All Fields] OR (""patient harm""[MeSH Terms] OR (""patient""[All Fields] AND ""harm""[All Fields]) OR ""patient harm""[All Fields]) OR ""safety management""[MeSH Terms]" | 253,795 |
| 2 | "(""effect""[All Fields] OR ""effecting""[All Fields] OR ""effective""[All Fields] OR ""effectively""[All Fields] OR ""effectiveness""[All Fields] OR ""effectivenesses""[All Fields] OR ""effectives""[All Fields] OR ""effectivities""[All Fields] OR ""effectivity""[All Fields] OR ""effects""[All Fields] OR ""effectiv*""[All Fields])",Most Recent,,"""effect""[All Fields] OR ""effecting""[All Fields] OR ""effective""[All Fields] OR ""effectively""[All Fields] OR ""effectiveness""[All Fields] OR ""effectivenesses""[All Fields] OR ""effectives""[All Fields] OR ""effectivities""[All Fields] OR ""effectivity""[All Fields] OR ""effects""[All Fields] OR ""effectiv*""[All Fields]" | 11,683,631 |
| 1 | velibra,Most Recent,,"""velibra""[All Fields]" | 1 |
| **Pubmed - Date Run: 14/07/2025 04:59:50** | | |
| ID | Search | Hits |
| 6 | #1 AND #5 AND (#2 OR #3 OR #4),Most Recent,,"""velibra""[All Fields] AND (""studies""[All Fields] OR ""study""[All Fields] OR ""study s""[All Fields] OR ""studying""[All Fields] OR ""studys""[All Fields] OR (""research personnel""[MeSH Terms] OR (""research""[All Fields] AND ""personnel""[All Fields]) OR ""research personnel""[All Fields] OR ""researcher""[All Fields] OR ""researchers""[All Fields] OR ""research""[MeSH Terms] OR ""research""[All Fields] OR ""research s""[All Fields] OR ""researchable""[All Fields] OR ""researche""[All Fields] OR ""researched""[All Fields] OR ""researcher s""[All Fields] OR ""researches""[All Fields] OR ""researching""[All Fields] OR ""researchs""[All Fields]) OR (""clinical trial""[Publication Type] OR ""clinical trials as topic""[MeSH Terms] OR ""clinical trial""[All Fields]) OR (""randomized controlled trial""[Publication Type] OR ""randomized controlled trials as topic""[MeSH Terms] OR ""randomized controlled trial""[All Fields] OR ""randomised controlled trial""[All Fields])) AND (""effect""[All Fields] OR ""effecting""[All Fields] OR ""effective""[All Fields] OR ""effectively""[All Fields] OR ""effectiveness""[All Fields] OR ""effectivenesses""[All Fields] OR ""effectives""[All Fields] OR ""effectivities""[All Fields] OR ""effectivity""[All Fields] OR ""effects""[All Fields] OR ""effectiv*""[All Fields] OR (""patient safety""[MeSH Terms] OR (""patient""[All Fields] AND ""safety""[All Fields]) OR ""patient safety""[All Fields] OR (""patient harm""[MeSH Terms] OR (""patient""[All Fields] AND ""harm""[All Fields]) OR ""patient harm""[All Fields]) OR ""safety management""[MeSH Terms]) OR (""equities""[All Fields] OR ""equity""[All Fields] OR ""disparit*""[All Fields] OR ""inequit*""[All Fields] OR ""inequalit*""[All Fields]))" | 1 |
| 5 | "(""studies""[All Fields] OR ""study""[All Fields] OR ""study s""[All Fields] OR ""studying""[All Fields] OR ""studys""[All Fields] OR (""research personnel""[MeSH Terms] OR (""research""[All Fields] AND ""personnel""[All Fields]) OR ""research personnel""[All Fields] OR ""researcher""[All Fields] OR ""researchers""[All Fields] OR ""research""[MeSH Terms] OR ""research""[All Fields] OR ""research s""[All Fields] OR ""researchable""[All Fields] OR ""researche""[All Fields] OR ""researched""[All Fields] OR ""researcher s""[All Fields] OR ""researches""[All Fields] OR ""researching""[All Fields] OR ""researchs""[All Fields]) OR (""clinical trial""[Publication Type] OR ""clinical trials as topic""[MeSH Terms] OR ""clinical trial""[All Fields]) OR (""randomized controlled trial""[Publication Type] OR ""randomized controlled trials as topic""[MeSH Terms] OR ""randomized controlled trial""[All Fields] OR ""randomised controlled trial""[All Fields])",Most Recent,,"""studies""[All Fields] OR ""study""[All Fields] OR ""study s""[All Fields] OR ""studying""[All Fields] OR ""studys""[All Fields] OR (""research personnel""[MeSH Terms] OR (""research""[All Fields] AND ""personnel""[All Fields]) OR ""research personnel""[All Fields] OR ""researcher""[All Fields] OR ""researchers""[All Fields] OR ""research""[MeSH Terms] OR ""research""[All Fields] OR ""research s""[All Fields] OR ""researchable""[All Fields] OR ""researche""[All Fields] OR ""researched""[All Fields] OR ""researcher s""[All Fields] OR ""researches""[All Fields] OR ""researching""[All Fields] OR ""researchs""[All Fields]) OR (""clinical trial""[Publication Type] OR ""clinical trials as topic""[MeSH Terms] OR ""clinical trial""[All Fields]) OR (""randomized controlled trial""[Publication Type] OR ""randomized controlled trials as topic""[MeSH Terms] OR ""randomized controlled trial""[All Fields] OR ""randomised controlled trial""[All Fields])" | 23,805,719 |
| 4 | "(""equities""[All Fields] OR ""equity""[All Fields] OR ""disparit*""[All Fields] OR ""inequit*""[All Fields] OR ""inequalit*""[All Fields])",Most Recent,,"""equities""[All Fields] OR ""equity""[All Fields] OR ""disparit*""[All Fields] OR ""inequit*""[All Fields] OR ""inequalit*""[All Fields]" | 317,276 |
| 3 | (""patient safety""[MeSH Terms] OR (""patient""[All Fields] AND ""safety""[All Fields]) OR ""patient safety""[All Fields] OR (""patient harm""[MeSH Terms] OR (""patient""[All Fields] AND ""harm""[All Fields]) OR ""patient harm""[All Fields]) OR ""safety management""[MeSH Terms])",Most Recent,,"""patient safety""[MeSH Terms] OR (""patient""[All Fields] AND ""safety""[All Fields]) OR ""patient safety""[All Fields] OR (""patient harm""[MeSH Terms] OR (""patient""[All Fields] AND ""harm""[All Fields]) OR ""patient harm""[All Fields]) OR ""safety management""[MeSH Terms]" | 278,587 |
| 2 | "(""effect""[All Fields] OR ""effecting""[All Fields] OR ""effective""[All Fields] OR ""effectively""[All Fields] OR ""effectiveness""[All Fields] OR ""effectivenesses""[All Fields] OR ""effectives""[All Fields] OR ""effectivities""[All Fields] OR ""effectivity""[All Fields] OR ""effects""[All Fields] OR ""effectiv*""[All Fields])",Most Recent,,"""effect""[All Fields] OR ""effecting""[All Fields] OR ""effective""[All Fields] OR ""effectively""[All Fields] OR ""effectiveness""[All Fields] OR ""effectivenesses""[All Fields] OR ""effectives""[All Fields] OR ""effectivities""[All Fields] OR ""effectivity""[All Fields] OR ""effects""[All Fields] OR ""effectiv*""[All Fields]" | 12,315,133 |
| 1 | velibra,Most Recent,,"""velibra""[All Fields]" | 2 |
| **Web of Science - Date Run: 30/07/2024 13:53:50** | | |
| ID | Search | Hits |
| 1 | ALL=(velibra) | 1 |
| 2 | ALL=((effect OR effective OR effectively OR effectiveness OR effects OR effectiv*)) | 15644528 |
| 3 | ALL=((patient safety OR patient harm OR safety management)) | 682985 |
| 4 | ALL=((equities OR equity OR disparit* OR inequit* OR inequalit*)) | 636039 |
| 5 | ALL=((studies OR study OR studying OR studys OR research personnel OR research OR researcher OR researchers OR clinical trial OR clinical trials as topic OR clinical trial OR randomized controlled trial OR randomized controlled trials as topic OR randomized controlled trial OR randomised controlled trial)) | 38704259 |
| 6 | #1 AND #5 AND (#2 OR #3 OR #4) | 1 |
| **Web of Science - Date Run: 14/07/2025 13:23:23** | | |
| ID | Search | Hits |
| 1 | ALL=(velibra) | 2 |
| 2 | ALL=(((effect OR effective OR effectively OR effectiveness OR effects OR effectiv*))) | 16777749 |
| 3 | ALL=((patient safety OR patient harm OR safety management)) | 758088 |
| 4 | ALL=((equities OR equity OR disparit* OR inequit* OR inequalit*)) | 713759 |
| 5 | ALL=((studies OR study OR studying OR studys OR research personnel OR research OR researcher OR researchers OR clinical trial OR clinical trials as topic OR clinical trial OR randomized controlled trial OR randomized controlled trials as topic OR randomized controlled trial OR randomised controlled trial)) | 41244021 |
| 6 | #1 AND #5 AND (#2 OR #3 OR #4) | 1 |
